# Clinical practice guideline for pediatric IgA vasculitis in Japan 2023: a digest-secondary publication

**DOI:** 10.1007/s10157-026-02859-0

**Published:** 2026-04-22

**Authors:** Masaki Shimizu, Kandai Nozu, Koichi Kamei, Satoru Arai, Shinichi Ansai, Toru Igarashi, Takashi Ishige, Maki Urushihara, Toshiyuki Ohta, Masafumi Oka, Yuko Shima, Keisuke Jimbo, Kazushi Tsuruga, Riku Hamada, Daishi Hirano, Takako Miyamae, Masaki Yamamoto, Yoshifusa Abe, Junichiro Araki, Naoko Ito, Chisato Umeda, Takayuki Okamoto, Shuya Kaneko, Yuji Kano, Tomohiro Kubota, Shohei Kuraoka, Satoko Kurata, Tomoyuki Sakai, Koji Sakuraya, Shunsuke Shinozuka, Wataru Shimabukuro, Toshihiko Shirakawa, Yoko Takagi, Hironori Takahashi, Maiko Tatsuki, Eriko Tanaka, Kazuki Tanaka, Chikako Terano, Kouki Tomari, Takeshi Ninchoji, Hideki Ban, Yuji Fujita, Kaori Fujiwara, Tomoko Horinouchi, Daisuke Matsuoka, Sohshi Matsumura, Takayuki Miyai, Saori Miwa, Miki Murakoshi, Junko Yasumura, Tomohiko Yamamura, Kazuna Yamamoto, Kazunari Kaneko

**Affiliations:** 1 Department of Pediatrics, Perinatal and Maternal Medicine, Graduate School of Medical and Dental Sciences, Institute of Science Tokyo, 1-5-45, Yushima, Bunkyo-Ku, Tokyo 113-8519 Japan; 2 Department of Pediatrics, Kobe University Graduate School of Medicine, 7-5-1 Kusunoki-Cho, Chuo, Kobe, Hyogo 650-0017 Japan; 3 Division of Nephrology and Rheumatology, National Center for Child Health and Development, 2-10-1, Okura, Setagaya-Ku, Tokyo 157-8535 Japan; 4 Department of Dermatology, St. Luke’s International Hospital, 9-1 Akashi-Cho, Chuo-Ku, Tokyo 104-8560 Japan; 5 Department of Dermatology, Nippon Medical School Musashikosugi Hospital, 1-383, Kosugi-Cho, Nakahara-Ku, Kawasaki City, Kanagawa 211-8533 Japan; 6 Department of Pediatrics, Nippon Medical School, 1-1-5, Sendagi, Bunkyo-Ku, Tokyo 113-8603 Japan; 7 Department of Pediatrics, Gunma University Graduate School of Medicine, 3-39-15 Showa-Machi, Maebashi, Gunma 371-8511 Japan; 8 Department of Pediatrics, Institute of Health Biosciences, The Tokushima University Graduate School, 3-18-15, Kuramoto, Tokushima 770-8503 Japan; 9 Department of Pediatric Nephrology, Hiroshima Prefectural Hospital, 1-5-54 Ujinakanda, Naka-Ku, Hiroshima, 730-0000 Japan; 10 Department of Pediatrics, Faculty of Medicine, Saga University, 5-1-1, Nabeshima, Saga 849-8501 Japan; 11 Department of Pediatrics, Wakayama Medical University, Kimiidera, Wakayama City, Wakayama 811-1 Japan; 12 Department of Pediatrics, Faculty of Medicine, Juntendo University, 2-1-1 Hongo, Bunkyo-Ku, Tokyo 113-8421 Japan; 13 Department of Pediatrics, NHOI Hirosaki General Medical Center, Tomino-Cho, Hirosaki-Shi, Aomori 036-8545 Japan; 14 Department of Nephrology and Rheumatology Tokyo Metropolitan Children’s Medical Center, 2-8-29 Musashidai, Fuchu, Tokyo 183-8561 Japan; 15 Department of Pediatrics, The Jikei University School of Medicine, 3-25-8, Nishi-Shimbashi, Minato-Ku, Tokyo 105-8461 Japan; 16 Department of Pediatric Rheumatology, Institute of Rheumatology, Tokyo Women’s Medical University Hospital, 8-1 Kawada-Cho, Shinjuku-Ku, Tokyo 162-8666 Japan; 17 Department of Pediatrics, Seirei Hamamatsu General Hospital, 2-12-12, Sumiyoshi, Chuo-Ku, Hamamatsu, Shizuoka 430-8558 Japan; 18 Children’s Medical Center, Showa Medical University Koto Toyosu Hospital, 5-1-38, Toyosu, Koto-Ku, Tokyo 135-8577 Japan; 19 Department of Pediatrics and Child Health, Kurume University School of Medicine, 67 Asahimachi, Kurume, 830-0011 Japan; 20 Department of Surgical Pathology, Tokyo Women’s Medical University, 8-1 Kawada-Cho, Shinjuku-Ku, Tokyo, 162-8666 Japan; 21 Department of Pediatrics, Hokkaido University Graduate School of Medicine, North 15, West 7, Sapporo, Hokkaido 060-8638 Japan; 22 Department of Pediatrics, Dokkyo Medical University, 880, Kitakobayashi, Mibu, Shimotsuga, Tochigi 321-0293 Japan; 23 Department of Pediatrics, Kagoshima Prefectural Satsunan Hospital, 4-11, Kaseda-Murahara, Minami-Satsuma City, Kagoshima 897-0001 Japan; 24 Department of Pediatrics, Faculty of Life Sciences, Kumamoto University, 1-1-1 Honjo, Kumamoto, 860-8556 Japan; 25 Department of Pediatrics, Shiga University of Medical Science, Setatsukinowa-Cho, Otsu, Shiga 525-0050 Japan; 26 Division of Nephrology, Saitama Children’s Medical Center, 1-2 Shin-Toshin, Chuo-Ku, Saitama City, Saitama 330-8777 Japan; 27 Department of Pediatrics, Matsudo City General Hospital, Children Medical Center, 993-1, Sendahori, Matsudo-Shi, Chiba 270-2296 Japan; 28 Department of Child Health and Welfare (Pediatrics), Graduate School of Medicine, University of the Ryukyus, 1076, Kiyuna, Ginowan-Shi, Okinawa 901-2720 Japan; 29 Department of Pediatrics, Nagasaki University Hospital, 1-7-1, Sakamoto, Nagasaki, 852-8501 Japan; 30 Department of Pediatrics, Asahikawa-Kosei General Hospital, 1-24-111, Asahikawa, Hokkaido Japan; 31 Department of Pediatrics, Kyorin University School of Medicine, 6-20-2 Shinkawa, Mitaka, Tokyo 181-8611 Japan; 32 Department of Nephrology, Aichi Children’s Health and Medical Center, 7-426 Morioka-Cho Oobu, Aichi, 474-8710 Japan; 33 Department of Pediatrics, Kitasato University School of Medicine, 1-15-1 Kitazato, Minami-Ku, Sagamihara, Kanagawa 252-0329 Japan; 34 Department of General Pediatrics, Okinawa Prefectural Nanbu Medical Center and Children’s Medical Center, 118-1 Arakawa, Haebaru-Cho, Okinawa, 901-1193 Japan; 35 Department of Pediatrics, Harima-Himeji General Medical Center, Kamiyacho 3-264, Himeji, Hyogo Japan; 36 Department of Pediatric Nephrology, Japanese Red Cross Kumamoto Hospital, 2-1-1 Nagamineminami, Higashi-Ku, Kumamoto, 861-8520 Japan; 37 Department of Pediatric Nephrology and Metabolism, Osaka Women’s and Children’s Hospital, 840 Murodo-Cho, Izumi, Osaka 594-1101 Japan; 38 Department of Pediatrics, Shinshu University School of Medicine, 3-1-1 Asahi, Matsumoto, Nagano 390-8621 Japan; 39 Department of Nephrology, Kanagawa Children’s Medical Center, 2-138-4 Mutsukawa, Minami-Ku, Yokohama, Kanagawa 232-8555 Japan; 40 Department of Pediatrics, Kagawa Prefectural Central Hospital, 1-2-1 Asahi-Machi, Takamatsu, Kagawa 760-8557 Japan; 41 Department of Pediatrics, Hiroshima Prefectural Hospital Organization Futabanosato Prefectural Hospital, 3-1-36 Futabanosato Higashi-Ku , Hiroshima-Shi, Hiroshima-Ken 732-0057 Japan; 42 Department of Pediatrics, Kansai Medical University, 2-5-1 Shin-Machi, Hirakata-Shi, Osaka 573-1010 Japan

**Keywords:** IgA vasculitis, IgA vasculitis nephritis, Guideline

## Introduction

IgA vasculitis (IgAV) is a common childhood vasculitis characterized by three key symptoms: skin, joint, and abdominal symptoms. This disease is frequently encountered in general pediatric practice. Approximately 30% of IgAV cases develop IgAV nephritis (IgAVN). It is a significant complication that determines the long-term prognosis of the patients. Severe IgAVN cases require evaluation through renal biopsy, and the condition’s severity is important for determining the subsequent treatment strategies. However, Japan currently has no clinical guidelines for pediatric IgAV. In response to a request for their creation, the Japanese Society of Pediatric Nephrology collaborated with the Japanese Society of Nephrology, the Japanese Society of Pediatric Rheumatology, the Japanese Society of Pediatric Gastroenterology and Hepatology, the Japanese Society of Pediatric Dermatology, and the Japanese Society of Dermatological Histopathology to develop these guidelines, which are Japan’s first clinical guidelines for pediatric IgAV, to clarify the basic treatment strategies based on accumulated evidence and to support the provision of high-quality, evidence-based medical care throughout Japan. This article provides a digest version of the 2023 Clinical Guidelines for Pediatric IgAV (in Japanese) (Shindan To Chiryo Sha, Inc., Tokyo, Japan) as a secondary publication in English, with a focus on key points [1, 2].Please verify if the provided city and country are correct and amend if necessary.We corrected the postal number of the Kansai Medical University.

### Guideline development process

The development of these guidelines required the organization of a guideline steering committee, a guideline development team, and a systematic review team. The committee members included pediatric nephrologists, pediatric rheumatologists, pediatric gastroenterologists, pediatric dermatologists, and skin histopathologists, all specialists in fields related to pediatric IgAV. Nephrologists, general pediatricians, and patients’ guardians also served as external reviewers, providing their opinions. For the systematic review, the keywords for each clinical question (CQ) were defined according to the scope. The search criteria were then developed at the request of the International Medical Information Center, a general incorporated foundation. Subsequently, a comprehensive and systematic literature search was conducted (Supplementary Table 1). Based on the disease frequency, the search primarily used PubMed as the database. The search period extended from January 1, 1966, to July 31, 2021. However, new literature was published during the compilation process; hence, the subsequent literature was also evaluated as necessary. Hand searches were conducted as necessary, with additional literature being included as deemed necessary. As a general rule, peer-reviewed articles that were in English and Japanese were selected.

Pediatric IgAV is a relatively common disease; however, intervention studies in children are scarce, and evidence-based management methods are limited. Therefore, CQ recommendations were limited to pharmacological therapies for which the level of evidence could be determined. For other treatments, the recommendations were written in a narrative format. For the items written in CQ format, the statement and strength of the recommendation grade (Table 1), along with the overall strength of the evidence (Table 2), were presented at the beginning. The gist of the recommendation was explained in the commentary based on the evidence accumulated. The strength of the recommendation and the overall strength of the evidence were determined by a vote among the guideline development committee. A consensus of 70% or more was set as the criterion for adoption.

**Table 1:** Strength of recommendation

1	We recommend
2	We suggest (weakly recommend)

**Table 2:** Strength of the total body of evidence

A(High)	We are confident that the true effect is close to the estimate of the effect
B(Moderate)	The true effect is likely to be closed to the estimate of the effect, but there is a possibility that it is substantially different
C(Low)	The true effect may be substantially different from the estimate of the effect
D(Very low)	The estimate of the effect is very uncertain, and often it will be far from the true effect

For CQs that did not reach a 70% consensus, a re-examination was held, and voting was done again until the required consensus was achieved. In February 2023, the final draft underwent external evaluation by the Japanese Society of Nephrology, which solicited public comments. The draft was revised as necessary and finalized.

### Guideline structure

The guidelines covered the following topics: concept and pathology of pediatric IgAV, epidemiology and prognosis, diagnosis, skin symptoms, skin pathology, arthritis symptoms, gastrointestinal symptoms, rare complications, general treatment overview, concept and pathology of pediatric IgAVN, diagnosis, clinical classification, pathological classification and their correlation with prognosis, and general treatment overview. CQs 1–14 were established regarding treatment. For six of these CQs, the evidence level could be determined. In these CQs, a statement, a recommendation grade, and an evidence summary and commentary were provided. For the remaining eight CQs, evidence summary and commentary were provided.

### Concept and pathogenesis of pediatric IgA vasculitis

IgAV is a common vasculitis seen in children. It is a small-scale vasculitis characterized by the deposition of IgA1-based immune complexes in the vessel walls. Palpable purpura is present, and leukocytoclastic vasculitis (LCV) is observed in the small vessels in the dermis.

### Epidemiology and prognosis

Ninety percent of pediatric patients with IgAV are under the age of 10. This disease is more prevalent in boys. It tends to be more prevalent in East Asia, including Japan. Compared to adult patients, the gastrointestinal symptoms in pediatric cases are more severe. Although there is a risk of recurrence and persistent renal complications, the prognosis for pediatric IgAV is generally good.

### Diagnosis of pediatric IgA vasculitis

The diagnosis of IgAV is primarily based on purpura. While the diagnosis is easy in typical cases, it can be somewhat difficult when other symptoms precede IgAV. Several classification criteria exist within the vasculitis syndrome, which are used as the diagnostic criteria (Tables 3 and 4) [3, 4].

**Table 3:** The American college of rheumatology 1990 criteria for the classification of Henoch-Schönlein purpura

%2． palpable purpura
%2． age ≤ 20 years at disease onset
%2． gastrointestinal bleeding
%2． biopsy showing granulocytes around arterioles or venules
The presence of any 2 or more of these criteria distinguish HSP from other forms of vasculitis

**Table 4:** EULAR/PRINTO/PRES criteria for Henoch-Schönlein purpura

A patient was classified as HSP in the presence of purpura or petechiae (mandatory) with lower limb predominance plus one of four criteria: (1) abdominal pain; (2) histopathology (IgA); (3) arthritis or arthralgia; (4) renal involvement	
Purpura	Purpura (commonly palpable and in crops) or petechiae, with lower limb predominance, not related to thrombocytopenia
Abdominal pain	Diffuse abdominal colicky pain with acute onset assessed by history and physical examination. May include intussusception and gastrointestinal bleeding
Histopathology	Typically leucocytoclastic vasculitis with predominant IgA deposit or proliferative glomerulonephritis with predominant IgA deposit
Arthritis or arthralgias	Arthritis of acute onset defined as joint swelling or joint pain with limitation on motion Arthralgia of acute onset defined as joint pain without joint swelling or limitation on motion
Renal involvement	Proteinuria > 0.3 g/24 h or > 30 mmol/mg of urine albumin/creatinine ratio on a spot morning sample Haematuria or red blood cell casts: > 5 red blood cells/high power field or red blood cells casts in the urinary sediment or ≥ 2 + on dipstick

### Skin symptoms

Skin symptoms are typical symptoms of IgAV. They are observed in almost all cases and frequently occur on the lower legs and the buttocks, presenting as multiple, raised, round, purpuric patches measuring a few to 10 mm in size. Joint and abdominal symptoms often occur simultaneously, and skin symptoms usually resolve without scarring within 4 weeks.

### Skin pathology

The histopathological features of skin lesions in IgAV are characterized by the infiltration of neutrophils, eosinophils, lymphocytes, and histiocytes with nuclear dust and extravasation of red blood cells around small blood vessels (mainly venules) in the upper to middle dermis. Fibrin deposition in some vascular walls and sometimes the surrounding areas is also observed. These features are typically seen in the peak stage of the rash (so-called palpable purpura). However, to demonstrate IgA deposition in vascular walls through immunofluorescence, rash specimens must be taken within 48–72 h of onset. However, in children with a typical skin rash, a skin biopsy is not required to diagnose IgAV.

### Joint symptoms

Joint symptoms such as pain and swelling, are one of the cardinal features of IgAV. They are observed in 50–80% of pediatric cases and most commonly affect the large joints of the lower limbs, such as the knees and the ankles. They usually resolve spontaneously within 1–5 days. In cases with severe joint pain or a limited range of motion, non-steroidal anti-inflammatory drugs (NSAIDs) or glucocorticoids (GCs) are recommended.

### Gastrointestinal symptoms

IgAV often presents with symptoms related to intestinal ischemia, such as abdominal pain, diarrhea, and bloody stools. These gastrointestinal symptoms are observed in 50–80% of pediatric cases. While most cases resolve spontaneously with symptomatic treatment, rare cases of intussusception and small bowel perforation require careful attention. Immunosuppressive therapy, including GCs, is recommended for refractory cases.

### Rare complications

IgAV is a systemic small-vessel vasculitis; hence, in addition to the three cardinal signs, other symptoms that are difficult to detect and severe multisystem complications have also been reported in severe cases [5–31]. During IgAV management, attention must be paid to neurological complications such as intracranial hemorrhage, acute encephalopathy, and reversible posterior leukoencephalopathy; respiratory complications such as diffuse alveolar hemorrhage; cardiovascular complications such as myocarditis, valvular disease, coronary artery aneurysm, and thromboembolism; gastrointestinal complications such as pancreatitis and cholecystitis; and genitourinary complications such as obstructive ureteritis and acute scrotum.

### Treatment of IgA vasculitis in children

**Fig. 1:**
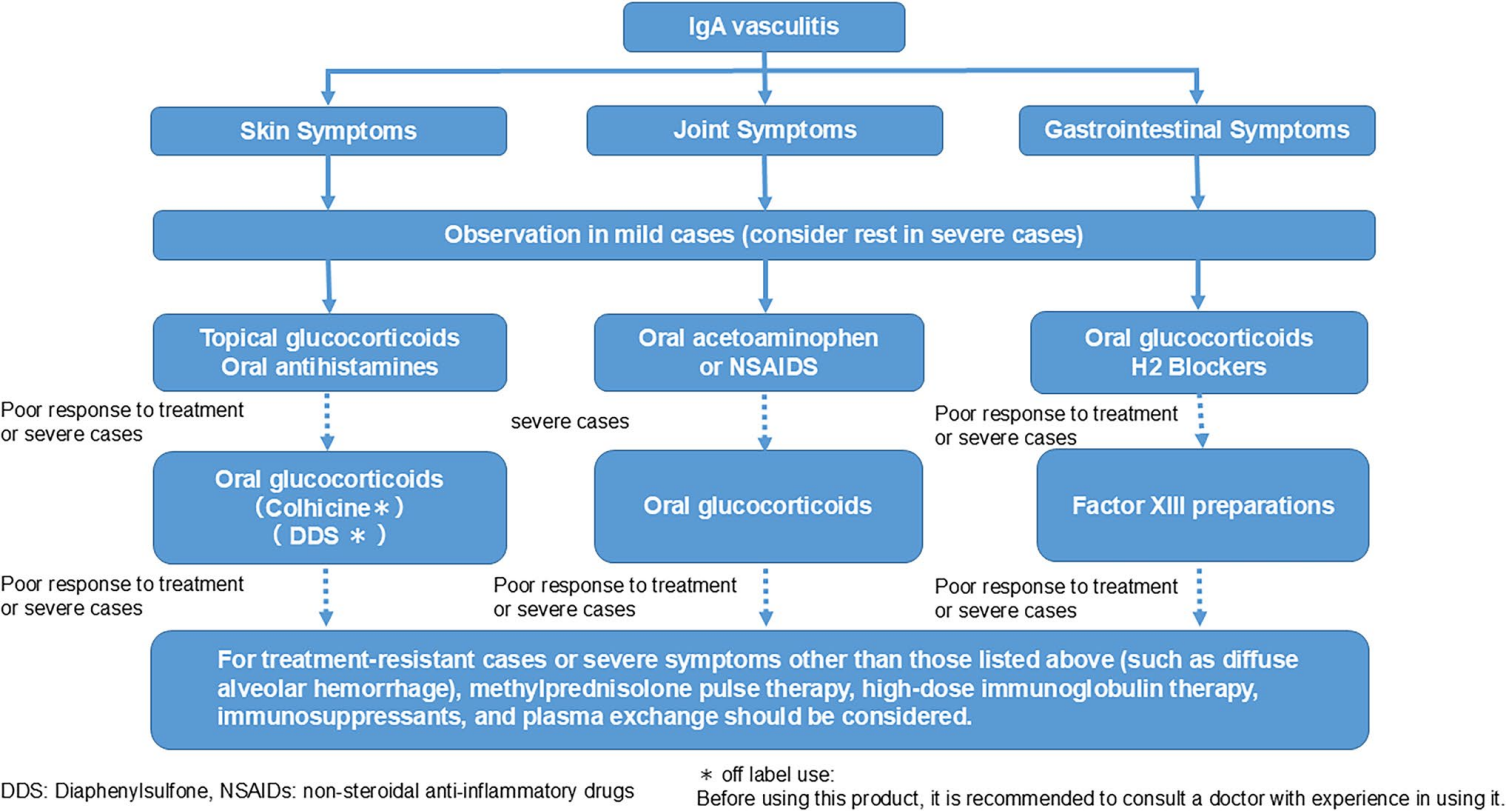
An outline of treatment for pediatric IgA vasculitis

IgAV treatment in children focuses on the treatment of each symptom, including the skin, joint, and abdominal symptoms. It is a self-limited disease; thus, mild cases improve with rest and short-term treatment. However, severe symptoms may require long-term use of GCs, and side effects must be closely monitored. Figure 1 depicts an outline of the treatment for childhood IgA vasculitis [1, 2].

### Concept and pathogenesis of IgA vasculitis nephritis

IgAVN is a nephropathy associated with IgAV, which occurs in 20–60% of patients within 4–6 weeks after the onset of IgAV. It is an important complication of IgAV that determines the long-term prognosis of patients with IgAV. Moreover, its pathogenesis involves glycosylated IgA1, similar to IgA nephropathy (IgAN) [32, 33].

### Diagnosis of IgA vasculitis nephritis

IgAVN is clinically diagnosed when IgAV is present, along with hematuria and proteinuria. A definitive diagnosis is made by performing a histopathological examination of a kidney biopsy. However, in clinical practice, IgAV is sometimes difficult to diagnose, and its differentiation from IgAN can be challenging. Currently, no solid evidence has been cited regarding the indications and timing of a renal biopsy [34].

### Clinical and pathological classification and their correlation with the prognosis

IgAVN is a secondary nephritis associated with IgAV. A kidney biopsy is not required for its diagnosis. The histology of a kidney biopsy for IgAVN is pathologically indistinguishable from IgAN. However, unlike IgAN, the severity of IgAVN tends to be determined early in the disease course, and the clinical and histopathological severities at the time of onset correlate with the kidney prognosis (Table 5) [35]. Therefore, treatment is determined by a kidney biopsy and a comprehensive assessment of the clinical symptoms and histopathological findings.

**Table 5:** Relationship between ISKDC histological grade, clinical features, and renal prognosis

ISKDC histological grade		Mesangial proliferation	Clinical features	Risk of chronic renal failure
I	Minimal change		Only hematuria	0%
II	Only Mesangial proliferation	Focal/Diffuse	Hematuria/proteinuria	< 5%
III	< 50% of glomeruli show crescentic/segmental lesions	Focal/Diffuse	Hematuria/proteinuria, acute nephritic syndrome, nephrotic syndrome	< 10%
IV	50–75%of glomeruli show crescentic/segmental lesions	Focal/Diffuse	Hematuria/proteinuria, acute nephritic syndrome, nephrotic syndrome, rapidly progressive nephritic syndrome	25%
V	≥ 75%of glomeruli show crescentic/segmental lesions	Focal/Diffuse	Hematuria/proteinuria, acute nephritic syndrome, nephrotic syndrome, rapidly progressive nephritic syndrome	> 50%
**VI**	Membranoproliferative nephritis-like	Focal/Diffuse		

### Treatment of pediatric IgA vasculitis nephritis

**Fig. 2:**
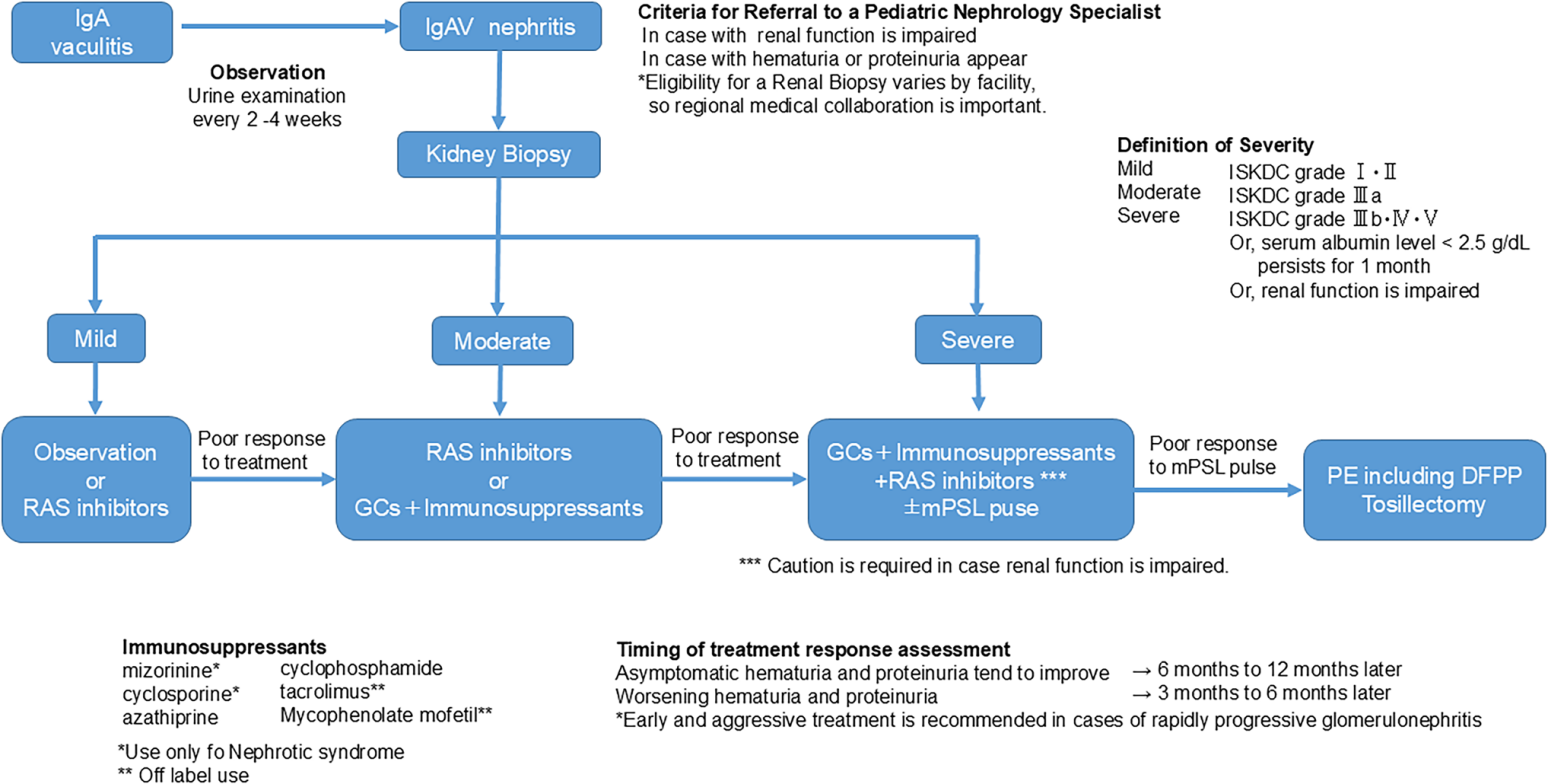
An outline of treatment for pediatric IgA vasculitis nephritis

IgAVN treatment is often determined by the classification of severity based on pathological findings. In severe cases, immunosuppressive therapy for acute lesions is the mainstay of treatment. For chronic lesions, renoprotective therapy with renin–angiotensin (RA) system inhibitors is selected. Nephritis tends to resolve spontaneously; hence, treatment should be tapered and discontinued once urinary findings normalize. However, in resistant or relapsing cases, the treatment needs to be intensified. The treatment aims to maintain long-term kidney function. To maintain long-term kidney function, it is important to eliminate residual proteinuria. Figure 2 presents an outline of IgAVN treatment [1, 2].

## CQ1: Is rest and exercise restriction recommended for pediatric patients with IgA vasculitis?

### Recommendations and statements

None.

#### Summary of evidence

There is no clinical trial evidence for rest and exercise restriction in pediatric and adult IgAV cases. In contrast, several textbooks and the literature describe rest and exercise restriction as viable management and treatment options. It is empirically known that rest and exercise restriction are effective in some cases, primarily for skin symptoms. However, there is little evidence regarding hospitalization for rest or restrictions on school activities, such as physical education, for children too young to rest at home.

Rest and exercise restriction are considered empirical treatments for recurrent skin and joint symptoms due to exercise, but excessive rest and exercise restriction can contribute to stress and muscle weakness. Thus, they should be kept to a minimum based on a patient’s condition and treatment effectiveness.

## CQ2: Is the administration of hemostatic agents and vasodilators recommended for pediatric patients with IgA vasculitis?

### Recommendation and Statement

None.

#### Summary of evidence

There is little evidence regarding the effectiveness of hemostatic agents and vasodilators for pediatric patients with IgAV. In contrast, no evidence has been presented to prohibit the administration of traditionally used hemostatic and vascular strengthening drugs (e.g., carbazochrome sodium sulfonate hydrate, tranexamic acid, and ascorbic acid) in the initial treatment of purpura, including that mediated by primary care physicians. Therefore, if these drugs can be confirmed to alleviate anxiety among patients and their parents and not lead to serious side effects, their use might be medically acceptable.

## CQ3: Are non-steroidal anti-inflammatory drugs or glucocorticoids recommended for joint symptoms in pediatric patients with IgA vasculitis?

### Recommendation and statement

Recommendation Grade D

For joint symptoms in pediatric patients with IgAV: (1) the use of NSAIDs may be considered, and (2) we recommend not using GCs; however, their use may be considered if severe joint symptoms are present.

#### Evidence summary

Although NSAIDs are commonly used for arthritis in pediatric IgAV, no studies worthy of evidence evaluation are available. The randomized controlled trials (RCTs) examining the efficacy of GCs have confirmed that prednisolone (PSL) improves the severity of joint symptoms, but its effectiveness in shortening the disease duration remains unclear [36, 37]. However, side effects such as weight gain and elevated blood pressure are more frequent in GCs-treated patients compared to placebo-treated patients [36]. Therefore, the risks and benefits of their use must be considered.

## CQ4: Is the administration of glucocorticoids recommended for abdominal symptoms in pediatric patients with IgA vasculitis?

### Recommendation and statement

Recommendation Grade 2C

We propose administering GCs to pediatric patients with IgAV to rapidly resolve abdominal pain and reduce its intensity.

#### Evidence summary

In Japan, GCs are often used to treat abdominal symptoms, particularly abdominal pain, in pediatric patients with IgAV. However, no high-level evidence has yet demonstrated their effectiveness. Two RCTs and four retrospective observational studies compared the severities of abdominal symptoms between GC- and non-GC-treated groups [36, 38–42]. One RCT found no significant difference in the duration of abdominal pain between these two groups [38]. However, another RCT demonstrated a significant reduction in the duration of abdominal pain in the intervention group [36]. In this RCT, the abdominal pain intensity improved in the intervention group, but significant weight gain and increases in diastolic blood pressure were also observed [36]. Three observational studies showed a high rate of abdominal pain resolution within 24 h of initiating GCs administration in the intervention group [39–41]. One study showed that initiating GCs administration within 2 days of admission significantly reduced the risk of abdominal surgery [42]. However, the level of evidence is low; hence, careful consideration is needed on an individual case-by-case basis rather than uniformly administering GCs.

## CQ5: Is the administration of factor XIII preparations recommended for abdominal symptoms in pediatric patients with IgA vasculitis?

### Recommendations and statements

None.

#### Evidence summary

Two RCTs have demonstrated the efficacy of factor XIII preparations for abdominal symptoms in pediatric patients with IgAV [43, 44]. These studies were small-group comparative studies, but reported significant improvements in severity scores, including abdominal symptoms (abdominal pain and bloody stool), in the factor XIII treatment group compared to the non-treatment group [43, 44]. Although the level of evidence is low, other case reports and case series have also reported that factor XIII treatment is effective for abdominal symptoms in approximately 90% of cases in patients with IgAV [45–60]. These findings suggest that factor XIII treatment may be effective for abdominal symptoms in pediatric patients with IgAV and may be considered as a viable treatment option.

## CQ6: Is antiulcer medication recommended for abdominal symptoms in pediatric patients with IgA vasculitis?

### Recommendation and statement

Recommendation Grade 2D.

We recommend administering a histamine H2 receptor antagonist to treat abdominal pain and gastrointestinal bleeding in patients with IgAV.

#### Evidence summary

An RCT evaluating antiulcer drugs for abdominal symptoms in pediatric IgAV reported that the histamine H2 receptor antagonist (H2RA) ranitidine group significantly reduced the duration of abdominal pain and gastrointestinal bleeding compared to the placebo group [61]. However, no RCTs in Japan have yet reported on the antiulcer drugs approved for pediatric use. Based on these findings, H2RA administration may be effective for abdominal pain and gastrointestinal bleeding in IgAV, and we recommend its use. However, the level of evidence is low.

**CQ7: What additional treatments are available for pediatric patients with severe/refractory IgA vasculitis who are not adequately responsive to glucocorticoids (excluding nephritis)?**

### Recommendations and statements

None.

#### Evidence summary

The following treatments have been reported when GCs are ineffective: methylprednisolone (mPSL) pulse therapy [62, 63], intravenous immunoglobulin (IVIG) [64–70], immunosuppressants [12, 22, 71–86], and plasma exchange [87–94]. None of these treatments is supported by sufficient evidence. Reports demonstrating the efficacy of these agents are limited to case reports without clear criteria for their initiation. However, in severely refractory patients, additional treatment might improve the outcomes. Careful selection based on the efficacy and side effects of each treatment may be necessary.

## CQ8: Are glucocorticoids recommended for the prevention of pediatric IgA vasculitis nephritis?

### Recommendations and statements

Recommendation Grade 1B.

We recommend against administering GCs to pediatric patients with IgAV as a preventative measure for IgAVN.

#### Evidence summary

Three RCTs examined the efficacy of GCs for preventing IgAVN in children with IgAV [36, 38, 95]. However, none of these studies demonstrated a preventive effect of GCs against IgAVN. An additional analysis in one of these RCTs found no difference in the long-term renal outcomes [96]. Based on these findings, we recommend not administering GCs to children with IgAV as a preventative measure against childhood purpura nephritis.

## CQ9: Are renin–angiotensin system inhibitors recommended for pediatric IgA vasculitis nephritis?

### Recommendation and statement

None.

#### Evidence summary

The RA system inhibitors are already widely used clinically for pediatric IgAVN; however, no RCTs or prospective studies examining the efficacy of RA system inhibitors alone have yet to be reported. Although most observational studies have reported the combination of RA system inhibitors with immunosuppressants or GCs, a small number of studies have shown that early administration of RA system inhibitors contributes to reduced proteinuria and improved renal prognosis [97–99]. The Kidney Disease Improving Global Outcomes guidelines of 2021 recommend the use of angiotensin-converting enzyme inhibitors or angiotensin II receptor blockers for pediatric cases of IgAVN with persistent proteinuria for 3 months or more [100]. These findings show that although the evidence level is not high, the administration of RA system inhibitors for pediatric IgAVN with persistent proteinuria might slow the progression of renal dysfunction and reduce proteinuria.

## CQ10: Are glucocorticoids and immunosuppressants recommended for severe cases of pediatric IgA vasculitis nephritis?

### Recommendations and statements

No recommendation grade: GCs monotherapy.

Recommendation grade 2B: Tacrolimus (TAC).

Recommendation grade 2C: Cyclophosphamide (CPA), azathioprine, cyclosporine (CyA), mycophenolate mofetil, and combination therapy.

The usage of GCs and immunosuppressants is recommended for severe pediatric IgAVN cases. However, there is little evidence supporting the efficacy of GC monotherapy in these cases. Accordingly, we suggest the usage of a combination of GCs and immunosuppressants.

#### Evidence summary

Regarding the usage of immunosuppressants in severe pediatric IgAVN cases, RCTs have been conducted on CPA [101], CyA [102], and TAC [103]. Several studies have used GCs in combination, making it difficult to conclude that immunosuppressants alone are effective. However, CyA monotherapy results in a high remission rate, and TAC monotherapy increases the remission rates and reduces the recurrence rates.

While reports demonstrating the efficacy of GC monotherapy are limited to observational studies, and the level of evidence is low, reports regarding combination therapy are limited to single-arm observational studies, and no high-level evidence has yet been made available. However, most observational studies examining the efficacy of immunosuppressants have used GCs in combination; hence, we conclude that the combination of GCs and immunosuppressants, while weak, may provide a basis for recommending its usage.

## CQ11: Is methylprednisolone pulse therapy recommended for severe cases of pediatric IgA vasculitis nephritis?

### Recommendations and statements

Recommendation Grade 2C.

mPSL pulse therapy may be effective and considered for severe cases of pediatric IgAVN.

#### Evidence summary

Only one RCT has examined the efficacy of mPSL pulse therapy for pediatric IgAVN. Subsequent long-term observational studies have shown that mPSL pulse therapy, along with immunosuppressants, may be effective in eliminating proteinuria, improving histological findings, and preserving renal function [102, 104]. In addition, small-scale retrospective studies have reported that mPSL pulse therapy is effective in improving proteinuria in early treatment after disease onset and in moderate pediatric IgAVN [105, 106].

## CQ12: Is pulse urokinase therapy recommended for severe cases of pediatric IgAVN?

### Recommendations and statements

None.

#### Evidence summary

Pulse urokinase therapy may be effective in reducing proteinuria in severe pediatric IgAVN cases; however, no prospective studies have yet examined its effectiveness. Several case reports and retrospective observational studies have been published in Japan, with some showing that pulse urokinase therapy is effective in improving proteinuria, hematuria, and histological findings [107–109]. Therefore, pulse urokinase therapy may be considered as a viable treatment option.

## CQ13: Is plasma exchange recommended for severe cases of pediatric IgA vasculitis nephritis?

### Recommendations and statements

None.

#### Evidence summary

Although there are no RCTs or prospective studies examining the efficacy of plasma exchange (PE) in severe histopathological and clinical cases of IgAVN, including children and adults, several small retrospective studies have reported that PE administered early in the course of the disease is effective and results in improved renal function and reduced urinary protein [91, 110–114]. Furthermore, the American Society for Apheresis guidelines classify PE for rapidly progressive crescentic IgAVN under a Grade 2C, Category III recommendation [115]. Based on these findings, PE administered early in the course of the disease may be effective in treating severe IgAVN cases (patients with rapidly progressive glomerulonephritis, nephrotic syndrome, or high incidence of cellular crescents).

## CQ14: Is the combination of tonsillectomy and methylprednisolone pulse therapy recommended for severe cases of pediatric IgA vasculitis nephritis?

### Recommendations and statements

None.

#### Evidence summary

No high-level evidence reports (e.g., RCTs or prospective interventional studies) have yet demonstrated the efficacy of the combination of tonsillectomy and mPSL pulse therapy (tonsillectomy pulse therapy) for pediatric IgAVN. This evidence is limited to retrospective observational studies from Japan [116–118]. Tonsillectomy pulse therapy for severe pediatric IgAVN cases is suggested to shorten the time to the resolution of proteinuria and to contribute to preventing recurrence, thereby making it a potential treatment option.

## Conclusion

The guidelines presented herein comprise a digest version of the 2023 Pediatric IgA Vasculitis Management Guidelines as a secondary publication in English, with a focus on the key points. IgAV is a disease encountered relatively frequently in daily clinical practice; however, evidence regarding its treatment is limited. As the guidelines undergo revisions, we hope that research will be conducted to address the clinical issues identified by the current guidelines, including the criteria for the need for rest, the determination of the appropriate timing and indications for kidney biopsy, and additional treatments for severe and relapsing cases with IgAV and IgAVN.

## Supplementary Information

Below is the link to the electronic supplementary material.

Supplementary file1 (DOCX 63 KB)
