# Supplementary material for: Clinical practice guideline for pediatric IgA vasculitis in Japan 2023: a digest-secondary publication
Source: Clin Exp Nephrol. 2026 Apr 22;30(7):959–72. doi: 10.1007/s10157-026-02859-0 (PMC13291043; doi:10.1007/s10157-026-02859-0)
Supplement: Supplementary file 1 — Supplementary file1 (DOCX 63 KB) [file 10157_2026_2859_MOESM1_ESM.docx]

**Supplement Table 1.　PubMed search formula and search results**

The search period was from January 1, 1966 to October 31, 2021, and the language was limited to English.

**CQ1: Is rest and exercise restriction recommended for pediatric patients with IgA vasculitis?**

Search date：22-Nov-2021

| No. | Search expression | Number of searches |
| --- | --- | --- |
| #1 | "purpura, schoenlein henoch"[MeSH Terms] OR ("purpura"[All Fields] AND "schoenlein henoch"[All Fields]) OR "schoenlein-henoch purpura"[All Fields] OR ("purpura"[All Fields] AND "schoenlein"[All Fields] AND "henoch"[All Fields]) OR "purpura schoenlein henoch"[All Fields] OR ("purpura, schoenlein henoch"[MeSH Terms] OR ("purpura"[All Fields] AND "schoenlein henoch"[All Fields]) OR "schoenlein-henoch purpura"[All Fields] OR ("purpura"[All Fields] AND "schonlein"[All Fields] AND "henoch"[All Fields]) OR "purpura schonlein henoch"[All Fields]) OR ("purpura, schoenlein henoch"[MeSH Terms] OR ("purpura"[All Fields] AND "schoenlein henoch"[All Fields]) OR "schoenlein-henoch purpura"[All Fields] OR "immunoglobulin a vasculitis"[All Fields]) OR ("IgA"[All Fields] AND ("vasculitide"[All Fields] OR "vasculities"[All Fields] OR "vasculitis"[MeSH Terms] OR "vasculitis"[All Fields] OR "vasculitides"[All Fields])) OR ("purpura, schoenlein henoch"[MeSH Terms] OR ("purpura"[All Fields] AND "schoenlein henoch"[All Fields]) OR "schoenlein-henoch purpura"[All Fields] OR ("allergic"[All Fields] AND "purpura"[All Fields]) OR "allergic purpura"[All Fields]) OR ("purpura, schoenlein henoch"[MeSH Terms] OR ("purpura"[All Fields] AND "schoenlein henoch"[All Fields]) OR "schoenlein-henoch purpura"[All Fields] OR ("anaphylactoid"[All Fields] AND "purpura"[All Fields]) OR "anaphylactoid purpura"[All Fields]) OR (("anaphylactic"[All Fields] OR "anaphylactically"[All Fields]) AND ("purpura"[MeSH Terms] OR "purpura"[All Fields] OR "purpuras"[All Fields])) | 6401 |
| #2 | rest[MeSH Terms] OR "rest"[All Fields] | 172,417 |
| #3 | exercise[MeSH Terms] OR "exercise"[All Fields] OR "exercises"[All Fields] OR "exercise therapy"[MeSH Terms] OR ("exercise"[All Fields] AND "therapy"[All Fields]) OR "exercise therapy"[All Fields] OR "exercise s"[All Fields] OR "exercised"[All Fields] OR "exerciser"[All Fields] OR "exercisers"[All Fields] OR "exercising"[All Fields] | 505,526 |
| #4 | exercise tolerance[MeSH Terms] OR ("exercise"[All Fields] AND "tolerance"[All Fields]) OR "exercise tolerance"[All Fields] | 26,695 |
| #5 | sport s[All Fields] OR "sports"[MeSH Terms] OR "sports"[All Fields] OR "sport"[All Fields] OR "sporting"[All Fields] | 374,373 |
| #06 | motor activity[MeSH Terms] OR ("motor"[All Fields] AND "activity"[All Fields]) OR "motor activity"[All Fields] | 375,421 |
| #7 | gravitation[MeSH Terms] OR "gravitation"[All Fields] OR "gravities"[All Fields] OR "gravity"[All Fields] OR "gravity s"[All Fields] | 40,349 |
| #8 | pressure[MeSH] OR pressure-dependent OR pressure dependent | 215,062 |
| #9 | "compress"[All Fields] OR "compressed"[All Fields] OR "compresses"[All Fields] OR "compressibilities"[All Fields] OR "compressibility"[All Fields] OR "compressible"[All Fields] OR "compressing"[All Fields] OR "compression"[All Fields] OR "compression s"[All Fields] OR "compressions"[All Fields] OR "compressive"[All Fields] OR "compressively"[All Fields] | 185,704 |
| #10 | #1 AND (#2 OR #3 OR #4 OR #5 OR #6 OR #7 OR #8 OR #9) | 78 |
| #11 | management | 3,281,185 |
| #12 | cutaneous | 197,344 |
| #13 | #1 AND (#11 OR #12) | 1,110 |

**CQ2: Is the administration of hemostatic agents and vasodilators recommended for pediatric patients with IgA vasculitis?**

Search date：26-Nov-2021

| No. | Search expression | Number of searches |
| --- | --- | --- |
| #1 | "purpura, schoenlein henoch"[MeSH Terms] OR ("purpura"[All Fields] AND "schoenlein henoch"[All Fields]) OR "schoenlein-henoch purpura"[All Fields] OR ("purpura"[All Fields] AND "schoenlein"[All Fields] AND "henoch"[All Fields]) OR "purpura schoenlein henoch"[All Fields] OR ("purpura, schoenlein henoch"[MeSH Terms] OR ("purpura"[All Fields] AND "schoenlein henoch"[All Fields]) OR "schoenlein-henoch purpura"[All Fields] OR ("purpura"[All Fields] AND "schonlein"[All Fields] AND "henoch"[All Fields]) OR "purpura schonlein henoch"[All Fields]) OR ("purpura, schoenlein henoch"[MeSH Terms] OR ("purpura"[All Fields] AND "schoenlein henoch"[All Fields]) OR "schoenlein-henoch purpura"[All Fields] OR "immunoglobulin a vasculitis"[All Fields]) OR ("IgA"[All Fields] AND ("vasculitide"[All Fields] OR "vasculities"[All Fields] OR "vasculitis"[MeSH Terms] OR "vasculitis"[All Fields] OR "vasculitides"[All Fields])) OR ("purpura, schoenlein henoch"[MeSH Terms] OR ("purpura"[All Fields] AND "schoenlein henoch"[All Fields]) OR "schoenlein-henoch purpura"[All Fields] OR ("allergic"[All Fields] AND "purpura"[All Fields]) OR "allergic purpura"[All Fields]) OR ("purpura, schoenlein henoch"[MeSH Terms] OR ("purpura"[All Fields] AND "schoenlein henoch"[All Fields]) OR "schoenlein-henoch purpura"[All Fields] OR ("anaphylactoid"[All Fields] AND "purpura"[All Fields]) OR "anaphylactoid purpura"[All Fields]) OR (("anaphylactic"[All Fields] OR "anaphylactically"[All Fields]) AND ("purpura"[MeSH Terms] OR "purpura"[All Fields] OR "purpuras"[All Fields])) | 6423 |
| #2 | hemostatics[Pharmacological Action] OR "hemostatics"[MeSH Terms] OR "hemostatics"[All Fields] OR ("hemostatic"[All Fields] AND "agent"[All Fields]) OR "hemostatic agent"[All Fields] | 143,140 |
| #3 | tranexamic acid[MeSH Terms] OR ("tranexamic"[All Fields] AND "acid"[All Fields]) OR "tranexamic acid"[All Fields] | 6,208 |
| #4 | "carbazochrome"[Supplementary Concept] OR "carbazochrome"[All Fields] | 76 |
| #5 | "ascorbic acid"[MeSH Terms] OR ("ascorbic"[All Fields] AND "acid"[All Fields]) OR "ascorbic acid"[All Fields] OR "vitamin c"[All Fields] OR "ascorb*"[All Fields] | 73,802 |
| #6 | #1 AND (#2 OR #3 OR #4 OR #5) | 33 |

**CQ3: Are non-steroidal anti-inflammatory drugs or glucocorticoids recommended for joint symptoms in pediatric patients with IgA vasculitis?**

Search date：24-Jan-2022

| No. | Search expression | Number of searches |
| --- | --- | --- |
| #1 | "purpura, schoenlein henoch"[MeSH Terms] OR ("purpura"[All Fields] AND "schoenlein henoch"[All Fields]) OR "schoenlein-henoch purpura"[All Fields] OR ("purpura"[All Fields] AND "schoenlein"[All Fields] AND "henoch"[All Fields]) OR "purpura schoenlein henoch"[All Fields] OR ("purpura, schoenlein henoch"[MeSH Terms] OR ("purpura"[All Fields] AND "schoenlein henoch"[All Fields]) OR "schoenlein-henoch purpura"[All Fields] OR ("purpura"[All Fields] AND "schonlein"[All Fields] AND "henoch"[All Fields]) OR "purpura schonlein henoch"[All Fields]) OR ("purpura, schoenlein henoch"[MeSH Terms] OR ("purpura"[All Fields] AND "schoenlein henoch"[All Fields]) OR "schoenlein-henoch purpura"[All Fields] OR "immunoglobulin a vasculitis"[All Fields]) OR ("IgA"[All Fields] AND ("vasculitide"[All Fields] OR "vasculities"[All Fields] OR "vasculitis"[MeSH Terms] OR "vasculitis"[All Fields] OR "vasculitides"[All Fields])) OR ("purpura, schoenlein henoch"[MeSH Terms] OR ("purpura"[All Fields] AND "schoenlein henoch"[All Fields]) OR "schoenlein-henoch purpura"[All Fields] OR ("allergic"[All Fields] AND "purpura"[All Fields]) OR "allergic purpura"[All Fields]) OR ("purpura, schoenlein henoch"[MeSH Terms] OR ("purpura"[All Fields] AND "schoenlein henoch"[All Fields]) OR "schoenlein-henoch purpura"[All Fields] OR ("anaphylactoid"[All Fields] AND "purpura"[All Fields]) OR "anaphylactoid purpura"[All Fields]) OR (("anaphylactic"[All Fields] OR "anaphylactically"[All Fields]) AND ("purpura"[MeSH Terms] OR "purpura"[All Fields] OR "purpuras"[All Fields])) | 4768 |
| #2 | "arthritis"[MeSH Terms] OR "arthritis"[All Fields] OR "arthritides"[All Fields] OR "polyarthritides"[All Fields] | 292355 |
| #3 | arthralgia"[MeSH Terms] OR "arthralgia"[All Fields] OR "arthralgias"[All Fields] | 19654 |
| #4 | arthralgia[MeSH Terms] OR "arthralgia"[All Fields] OR ("joint"[All Fields] AND "pain"[All Fields]) OR "joint pain"[All Fields] | 85706 |
| #5 | ("joint s"[All Fields] OR "joints"[MeSH Terms] OR "joints"[All Fields] OR "joint"[All Fields]) AND ("involve"[All Fields] OR "involved"[All Fields] OR "involvement"[All Fields] OR "involvements"[All Fields] OR "involves"[All Fields] OR "involving"[All Fields] OR "involvment"[All Fields]) | 55910 |
| #6 | "joint diseases"[MeSH Terms] | 306573 |
| #7 | #1 AND (#2 OR #3 OR #4 OR #5 OR #6) | 776 |
| #8 | "steroidal"[All Fields] OR "steroidals"[All Fields] OR "steroidic"[All Fields] OR "steroids"[MeSH Terms] OR "steroids"[All Fields] OR "steroid"[All Fields] OR ("glucocorticoids"[Pharmacological Action] OR "glucocorticoids"[MeSH Terms] OR "glucocorticoids"[All Fields] OR "glucocorticoid"[All Fields]) OR ("adrenal cortex hormones"[MeSH Terms] OR ("adrenal"[All Fields] AND "cortex"[All Fields] AND "hormones"[All Fields]) OR "adrenal cortex hormones"[All Fields] OR "corticosteroid"[All Fields] OR "corticosteroids"[All Fields] OR "corticosteroidal"[All Fields] OR "corticosteroide"[All Fields] OR "corticosteroides"[All Fields]) OR ("hydrocortisone"[MeSH Terms] OR "hydrocortisone"[All Fields] OR "hydrocortisones"[All Fields]) OR ("dexamethason"[All Fields] OR "dexamethasone"[MeSH Terms] OR "dexamethasone"[All Fields] OR "dexamethasone s"[All Fields] OR "dexamethasones"[All Fields]) OR ("prednison"[All Fields] OR "prednisone"[MeSH Terms] OR "prednisone"[All Fields]) OR ("prednisolon"[All Fields] OR "prednisolone"[MeSH Terms] OR "prednisolone"[All Fields]) OR ("methylprednisolone"[MeSH Terms] OR "methylprednisolone"[All Fields] OR "methylprednisolon"[All Fields]) OR ("adrenal cortex hormones"[MeSH Terms] OR ("adrenal"[All Fields] AND "cortex"[All Fields] AND "hormones"[All Fields]) OR "adrenal cortex hormones"[All Fields] OR ("adrenal"[All Fields] AND "cortex"[All Fields] AND "hormone"[All Fields]) OR "adrenal cortex hormone"[All Fields]) | 1059491 |
| #9 | "analgesics, non-narcotic"[MeSH Terms] OR "anti-inflammatory agents"[MeSH Terms] | 161607 |
| #10 | #7 AND (#8 OR #9) | 225 |

**CQ4: Is the administration of glucocorticoids recommended for abdominal symptoms in pediatric patients with IgA vasculitis?**

Search date：9-Nov-2021

| No. | Search expression | Number of searches |
| --- | --- | --- |
| #1 | "purpura, schoenlein henoch"[MeSH Terms] OR ("purpura"[All Fields] AND "schoenlein henoch"[All Fields]) OR "schoenlein-henoch purpura"[All Fields] OR ("purpura"[All Fields] AND "schoenlein"[All Fields] AND "henoch"[All Fields]) OR "purpura schoenlein henoch"[All Fields] OR ("purpura, schoenlein henoch"[MeSH Terms] OR ("purpura"[All Fields] AND "schoenlein henoch"[All Fields]) OR "schoenlein-henoch purpura"[All Fields] OR ("purpura"[All Fields] AND "schonlein"[All Fields] AND "henoch"[All Fields]) OR "purpura schonlein henoch"[All Fields]) OR ("purpura, schoenlein henoch"[MeSH Terms] OR ("purpura"[All Fields] AND "schoenlein henoch"[All Fields]) OR "schoenlein-henoch purpura"[All Fields] OR "immunoglobulin a vasculitis"[All Fields]) OR ("IgA"[All Fields] AND ("vasculitide"[All Fields] OR "vasculities"[All Fields] OR "vasculitis"[MeSH Terms] OR "vasculitis"[All Fields] OR "vasculitides"[All Fields])) OR ("purpura, schoenlein henoch"[MeSH Terms] OR ("purpura"[All Fields] AND "schoenlein henoch"[All Fields]) OR "schoenlein-henoch purpura"[All Fields] OR ("allergic"[All Fields] AND "purpura"[All Fields]) OR "allergic purpura"[All Fields]) OR ("purpura, schoenlein henoch"[MeSH Terms] OR ("purpura"[All Fields] AND "schoenlein henoch"[All Fields]) OR "schoenlein-henoch purpura"[All Fields] OR ("anaphylactoid"[All Fields] AND "purpura"[All Fields]) OR "anaphylactoid purpura"[All Fields]) OR (("anaphylactic"[All Fields] OR "anaphylactically"[All Fields]) AND ("purpura"[MeSH Terms] OR "purpura"[All Fields] OR "purpuras"[All Fields])) | 7128 |
| #2 | (("gastrointestinal"[All Fields] OR "gastrointestinally"[All Fields] OR "gastrointestine"[All Fields]) AND "symptom*"[All Fields]) OR (("gastrointestinal"[All Fields] OR "gastrointestinally"[All Fields] OR "gastrointestine"[All Fields]) AND ("manifest"[All Fields] OR "manifestating"[All Fields] OR "manifestation"[All Fields] OR "manifestations"[All Fields] OR "manifested"[All Fields] OR "manifesting"[All Fields] OR "manifestion"[All Fields] OR "manifestions"[All Fields] OR "manifests"[All Fields])) OR (("gastrointestinal"[All Fields] OR "gastrointestinally"[All Fields] OR "gastrointestine"[All Fields]) AND ("involve"[All Fields] OR "involved"[All Fields] OR "involvement"[All Fields] OR "involvements"[All Fields] OR "involves"[All Fields] OR "involving"[All Fields] OR "involvment"[All Fields])) OR (("gastrointestinal"[All Fields] OR "gastrointestinally"[All Fields] OR "gastrointestine"[All Fields]) AND ("pain"[MeSH Terms] OR "pain"[All Fields])) OR (("abdomen"[MeSH Terms] OR "abdomen"[All Fields] OR "abdominal"[All Fields] OR "abdominally"[All Fields] OR "abdominals"[All Fields]) AND "symptom*"[All Fields]) OR (("abdomen"[MeSH Terms] OR "abdomen"[All Fields] OR "abdominal"[All Fields] OR "abdominally"[All Fields] OR "abdominals"[All Fields]) AND ("manifest"[All Fields] OR "manifestating"[All Fields] OR "manifestation"[All Fields] OR "manifestations"[All Fields] OR "manifested"[All Fields] OR "manifesting"[All Fields] OR "manifestion"[All Fields] OR "manifestions"[All Fields] OR "manifests"[All Fields])) OR (("abdomen"[MeSH Terms] OR "abdomen"[All Fields] OR "abdominal"[All Fields] OR "abdominally"[All Fields] OR "abdominals"[All Fields]) AND ("involve"[All Fields] OR "involved"[All Fields] OR "involvement"[All Fields] OR "involvements"[All Fields] OR "involves"[All Fields] OR "involving"[All Fields] OR "involvment"[All Fields])) OR ("abdominal pain"[MeSH Terms] OR ("abdominal"[All Fields] AND "pain"[All Fields]) OR "abdominal pain"[All Fields]) OR ("stomachache"[All Fields] OR "stomachaches"[All Fields]) OR ("colic"[MeSH Terms] OR "colic"[All Fields] OR "colics"[All Fields]) OR ("vomiter"[All Fields] OR "vomiters"[All Fields] OR "vomiting"[MeSH Terms] OR "vomiting"[All Fields] OR "vomit"[All Fields] OR "vomited"[All Fields] OR "vomits"[All Fields] OR "vomitings"[All Fields] OR "vomition"[All Fields] OR "vomitting"[All Fields]) OR ("gastrointestinal hemorrhage"[MeSH Terms] OR ("gastrointestinal"[All Fields] AND "hemorrhage"[All Fields]) OR "gastrointestinal hemorrhage"[All Fields] OR ("bloody"[All Fields] AND "stool"[All Fields]) OR "bloody stool"[All Fields]) OR ("perforant"[All Fields] OR "perforants"[All Fields] OR "perforate"[All Fields] OR "perforated"[All Fields] OR "perforates"[All Fields] OR "perforating"[All Fields] OR "perforation"[All Fields] OR "perforations"[All Fields] OR "perforative"[All Fields] OR "perforator"[All Fields] OR "perforator s"[All Fields] OR "perforators"[All Fields]) OR ("gastrointestinal hemorrhage"[MeSH Terms] OR ("gastrointestinal"[All Fields] AND "hemorrhage"[All Fields]) OR "gastrointestinal hemorrhage"[All Fields] OR ("gastrointestinal"[All Fields] AND "bleeding"[All Fields]) OR "gastrointestinal bleeding"[All Fields]) OR (("abdomen"[MeSH Terms] OR "abdomen"[All Fields] OR "abdominal"[All Fields] OR "abdominally"[All Fields] OR "abdominals"[All Fields]) AND ("bleedings"[All Fields] OR "hemorrhage"[MeSH Terms] OR "hemorrhage"[All Fields] OR "bleed"[All Fields] OR "bleeding"[All Fields] OR "bleeds"[All Fields])) OR ("gastritis"[MeSH Terms] OR "gastritis"[All Fields] OR "gastritides"[All Fields]) OR ("duodenitis"[MeSH Terms] OR "duodenitis"[All Fields] OR "duodenum"[MeSH Terms] OR "duodenum"[All Fields] OR "duodenal"[All Fields]) OR (("periumbilical"[All Fields] OR "periumbilically"[All Fields]) AND ("pain"[MeSH Terms] OR "pain"[All Fields])) OR (("epigastric"[All Fields] OR "epigastrical"[All Fields]) AND ("pain"[MeSH Terms] OR "pain"[All Fields])) OR ("intussuscepted"[All Fields] OR "intussuscepting"[All Fields] OR "intussusception"[MeSH Terms] OR "intussusception"[All Fields] OR "intussusceptions"[All Fields]) OR ("nausea"[MeSH Terms] OR "nausea"[All Fields] OR "nauseas"[All Fields]) OR ("ulcer"[MeSH Terms] OR "ulcer"[All Fields] OR "ulcerate"[All Fields] OR "ulcerated"[All Fields] OR "ulcerates"[All Fields] OR "ulcerating"[All Fields] OR "ulceration"[All Fields] OR "ulcerations"[All Fields] OR "ulcerative"[All Fields] OR "ulcers"[All Fields] OR "ulcer s"[All Fields] OR "ulcerous"[All Fields]) | 849202 |
| #3 | signs and symptoms, digestive[MeSH Terms] OR "gastrointestinal diseases"[MeSH Terms] | 1122470 |
| #4 | #1 AND (#2 OR #3 | 1699 |
| #5 | "steroidal"[All Fields] OR "steroidals"[All Fields] OR "steroidic"[All Fields] OR "steroids"[MeSH Terms] OR "steroids"[All Fields] OR "steroid"[All Fields] OR ("glucocorticoids"[Pharmacological Action] OR "glucocorticoids"[MeSH Terms] OR "glucocorticoids"[All Fields] OR "glucocorticoid"[All Fields]) OR ("adrenal cortex hormones"[MeSH Terms] OR ("adrenal"[All Fields] AND "cortex"[All Fields] AND "hormones"[All Fields]) OR "adrenal cortex hormones"[All Fields] OR "corticosteroid"[All Fields] OR "corticosteroids"[All Fields] OR "corticosteroidal"[All Fields] OR "corticosteroide"[All Fields] OR "corticosteroides"[All Fields]) OR ("hydrocortisone"[MeSH Terms] OR "hydrocortisone"[All Fields] OR "hydrocortisones"[All Fields]) OR ("dexamethason"[All Fields] OR "dexamethasone"[MeSH Terms] OR "dexamethasone"[All Fields] OR "dexamethasone s"[All Fields] OR "dexamethasones"[All Fields]) OR ("prednison"[All Fields] OR "prednisone"[MeSH Terms] OR "prednisone"[All Fields]) OR ("prednisolon"[All Fields] OR "prednisolone"[MeSH Terms] OR "prednisolone"[All Fields]) OR ("methylprednisolone"[MeSH Terms] OR "methylprednisolone"[All Fields] OR "methylprednisolon"[All Fields]) OR ("adrenal cortex hormones"[MeSH Terms] OR ("adrenal"[All Fields] AND "cortex"[All Fields] AND "hormones"[All Fields]) OR "adrenal cortex hormones"[All Fields] OR ("adrenal"[All Fields] AND "cortex"[All Fields] AND "hormone"[All Fields]) OR "adrenal cortex hormone"[All Fields]) | 1270562 |
| #6 | #4 AND #5 | 30770587 |
| 6 | 1966/01/01:2021/10/31[dp] | 30770587 |
| 7 | english[language] | 28515304 |
| 8 | #4 and #5 and #6 and #7 | 400 |

**CQ5: Is the administration of factor XIII preparations recommended for abdominal symptoms in pediatric patients with IgA vasculitis?**

Search date：17-Nov-2021

| No. | Search expression | Number of searches |
| --- | --- | --- |
| #1 | "purpura, schoenlein henoch"[MeSH Terms] OR ("purpura"[All Fields] AND "schoenlein henoch"[All Fields]) OR "schoenlein-henoch purpura"[All Fields] OR ("purpura"[All Fields] AND "schoenlein"[All Fields] AND "henoch"[All Fields]) OR "purpura schoenlein henoch"[All Fields] OR ("purpura, schoenlein henoch"[MeSH Terms] OR ("purpura"[All Fields] AND "schoenlein henoch"[All Fields]) OR "schoenlein-henoch purpura"[All Fields] OR ("purpura"[All Fields] AND "schonlein"[All Fields] AND "henoch"[All Fields]) OR "purpura schonlein henoch"[All Fields]) OR ("purpura, schoenlein henoch"[MeSH Terms] OR ("purpura"[All Fields] AND "schoenlein henoch"[All Fields]) OR "schoenlein-henoch purpura"[All Fields] OR "immunoglobulin a vasculitis"[All Fields]) OR ("IgA"[All Fields] AND ("vasculitide"[All Fields] OR "vasculities"[All Fields] OR "vasculitis"[MeSH Terms] OR "vasculitis"[All Fields] OR "vasculitides"[All Fields])) OR ("purpura, schoenlein henoch"[MeSH Terms] OR ("purpura"[All Fields] AND "schoenlein henoch"[All Fields]) OR "schoenlein-henoch purpura"[All Fields] OR ("allergic"[All Fields] AND "purpura"[All Fields]) OR "allergic purpura"[All Fields]) OR ("purpura, schoenlein henoch"[MeSH Terms] OR ("purpura"[All Fields] AND "schoenlein henoch"[All Fields]) OR "schoenlein-henoch purpura"[All Fields] OR ("anaphylactoid"[All Fields] AND "purpura"[All Fields]) OR "anaphylactoid purpura"[All Fields]) OR (("anaphylactic"[All Fields] OR "anaphylactically"[All Fields]) AND ("purpura"[MeSH Terms] OR "purpura"[All Fields] OR "purpuras"[All Fields])) | 7140 |
| #2 | (("gastrointestinal"[All Fields] OR "gastrointestinally"[All Fields] OR "gastrointestine"[All Fields]) AND "symptom*"[All Fields]) OR (("gastrointestinal"[All Fields] OR "gastrointestinally"[All Fields] OR "gastrointestine"[All Fields]) AND ("manifest"[All Fields] OR "manifestating"[All Fields] OR "manifestation"[All Fields] OR "manifestations"[All Fields] OR "manifested"[All Fields] OR "manifesting"[All Fields] OR "manifestion"[All Fields] OR "manifestions"[All Fields] OR "manifests"[All Fields])) OR (("gastrointestinal"[All Fields] OR "gastrointestinally"[All Fields] OR "gastrointestine"[All Fields]) AND ("involve"[All Fields] OR "involved"[All Fields] OR "involvement"[All Fields] OR "involvements"[All Fields] OR "involves"[All Fields] OR "involving"[All Fields] OR "involvment"[All Fields])) OR (("gastrointestinal"[All Fields] OR "gastrointestinally"[All Fields] OR "gastrointestine"[All Fields]) AND ("pain"[MeSH Terms] OR "pain"[All Fields])) OR (("abdomen"[MeSH Terms] OR "abdomen"[All Fields] OR "abdominal"[All Fields] OR "abdominally"[All Fields] OR "abdominals"[All Fields]) AND "symptom*"[All Fields]) OR (("abdomen"[MeSH Terms] OR "abdomen"[All Fields] OR "abdominal"[All Fields] OR "abdominally"[All Fields] OR "abdominals"[All Fields]) AND ("manifest"[All Fields] OR "manifestating"[All Fields] OR "manifestation"[All Fields] OR "manifestations"[All Fields] OR "manifested"[All Fields] OR "manifesting"[All Fields] OR "manifestion"[All Fields] OR "manifestions"[All Fields] OR "manifests"[All Fields])) OR (("abdomen"[MeSH Terms] OR "abdomen"[All Fields] OR "abdominal"[All Fields] OR "abdominally"[All Fields] OR "abdominals"[All Fields]) AND ("involve"[All Fields] OR "involved"[All Fields] OR "involvement"[All Fields] OR "involvements"[All Fields] OR "involves"[All Fields] OR "involving"[All Fields] OR "involvment"[All Fields])) OR ("abdominal pain"[MeSH Terms] OR ("abdominal"[All Fields] AND "pain"[All Fields]) OR "abdominal pain"[All Fields]) OR ("stomachache"[All Fields] OR "stomachaches"[All Fields]) OR ("colic"[MeSH Terms] OR "colic"[All Fields] OR "colics"[All Fields]) OR ("vomiter"[All Fields] OR "vomiters"[All Fields] OR "vomiting"[MeSH Terms] OR "vomiting"[All Fields] OR "vomit"[All Fields] OR "vomited"[All Fields] OR "vomits"[All Fields] OR "vomitings"[All Fields] OR "vomition"[All Fields] OR "vomitting"[All Fields]) OR ("gastrointestinal hemorrhage"[MeSH Terms] OR ("gastrointestinal"[All Fields] AND "hemorrhage"[All Fields]) OR "gastrointestinal hemorrhage"[All Fields] OR ("bloody"[All Fields] AND "stool"[All Fields]) OR "bloody stool"[All Fields]) OR ("perforant"[All Fields] OR "perforants"[All Fields] OR "perforate"[All Fields] OR "perforated"[All Fields] OR "perforates"[All Fields] OR "perforating"[All Fields] OR "perforation"[All Fields] OR "perforations"[All Fields] OR "perforative"[All Fields] OR "perforator"[All Fields] OR "perforator s"[All Fields] OR "perforators"[All Fields]) OR ("gastrointestinal hemorrhage"[MeSH Terms] OR ("gastrointestinal"[All Fields] AND "hemorrhage"[All Fields]) OR "gastrointestinal hemorrhage"[All Fields] OR ("gastrointestinal"[All Fields] AND "bleeding"[All Fields]) OR "gastrointestinal bleeding"[All Fields]) OR (("abdomen"[MeSH Terms] OR "abdomen"[All Fields] OR "abdominal"[All Fields] OR "abdominally"[All Fields] OR "abdominals"[All Fields]) AND ("bleedings"[All Fields] OR "hemorrhage"[MeSH Terms] OR "hemorrhage"[All Fields] OR "bleed"[All Fields] OR "bleeding"[All Fields] OR "bleeds"[All Fields])) OR ("gastritis"[MeSH Terms] OR "gastritis"[All Fields] OR "gastritides"[All Fields]) OR ("duodenitis"[MeSH Terms] OR "duodenitis"[All Fields] OR "duodenum"[MeSH Terms] OR "duodenum"[All Fields] OR "duodenal"[All Fields]) OR (("periumbilical"[All Fields] OR "periumbilically"[All Fields]) AND ("pain"[MeSH Terms] OR "pain"[All Fields])) OR (("epigastric"[All Fields] OR "epigastrical"[All Fields]) AND ("pain"[MeSH Terms] OR "pain"[All Fields])) OR ("intussuscepted"[All Fields] OR "intussuscepting"[All Fields] OR "intussusception"[MeSH Terms] OR "intussusception"[All Fields] OR "intussusceptions"[All Fields]) OR ("nausea"[MeSH Terms] OR "nausea"[All Fields] OR "nauseas"[All Fields]) OR ("ulcer"[MeSH Terms] OR "ulcer"[All Fields] OR "ulcerate"[All Fields] OR "ulcerated"[All Fields] OR "ulcerates"[All Fields] OR "ulcerating"[All Fields] OR "ulceration"[All Fields] OR "ulcerations"[All Fields] OR "ulcerative"[All Fields] OR "ulcers"[All Fields] OR "ulcer s"[All Fields] OR "ulcerous"[All Fields]) | 850177 |
| #3 | signs and symptoms, digestive[MeSH Terms] OR "gastrointestinal diseases"[MeSH Terms] | 1123530 |
| #4 | #1 AND (#2 OR #3) | 1701 |
| #5 | factor xiii[MeSH Terms] OR ("factor"[All Fields] AND "xiii"[All Fields]) OR "factor xiii"[All Fields] OR ("fibrinolysin"[MeSH Terms] OR "fibrinolysin"[All Fields] OR "fibrogammin"[All Fields]) OR ("cryoprecipitability"[All Fields] OR "cryoprecipitable"[All Fields] OR "cryoprecipitate"[All Fields] OR "cryoprecipitated"[All Fields] OR "cryoprecipitates"[All Fields] OR "cryoprecipitating"[All Fields] OR "cryoprecipitation"[All Fields]) OR ("fibrin stabilising factor"[All Fields] OR "factor xiii"[MeSH Terms] OR ("factor"[All Fields] AND "xiii"[All Fields]) OR "factor xiii"[All Fields] OR ("fibrin"[All Fields] AND "stabilizing"[All Fields] AND "factor"[All Fields]) OR "fibrin stabilizing factor"[All Fields]) OR ("Novo"[All Fields] AND "Thirteen"[All Fields]) | 17193 |
| #6 | "Factor 13"[All Fields] OR "laki lorand factor"[All Fields] OR "laki lorand factor"[All Fields] | 222 |
| #7 | #4 AND (#5 OR #6) | 36 |

**CQ6: Is antiulcer medication recommended for abdominal symptoms in pediatric patients with IgA vasculitis?**

Search date：9-Nov-2021

| No. | Search expression | Number of searches |
| --- | --- | --- |
| #1 | "purpura, schoenlein henoch"[MeSH Terms] OR ("purpura"[All Fields] AND "schoenlein henoch"[All Fields]) OR "schoenlein-henoch purpura"[All Fields] OR ("purpura"[All Fields] AND "schoenlein"[All Fields] AND "henoch"[All Fields]) OR "purpura schoenlein henoch"[All Fields] OR ("purpura, schoenlein henoch"[MeSH Terms] OR ("purpura"[All Fields] AND "schoenlein henoch"[All Fields]) OR "schoenlein-henoch purpura"[All Fields] OR ("purpura"[All Fields] AND "schonlein"[All Fields] AND "henoch"[All Fields]) OR "purpura schonlein henoch"[All Fields]) OR ("purpura, schoenlein henoch"[MeSH Terms] OR ("purpura"[All Fields] AND "schoenlein henoch"[All Fields]) OR "schoenlein-henoch purpura"[All Fields] OR "immunoglobulin a vasculitis"[All Fields]) OR ("IgA"[All Fields] AND ("vasculitide"[All Fields] OR "vasculities"[All Fields] OR "vasculitis"[MeSH Terms] OR "vasculitis"[All Fields] OR "vasculitides"[All Fields])) OR ("purpura, schoenlein henoch"[MeSH Terms] OR ("purpura"[All Fields] AND "schoenlein henoch"[All Fields]) OR "schoenlein-henoch purpura"[All Fields] OR ("allergic"[All Fields] AND "purpura"[All Fields]) OR "allergic purpura"[All Fields]) OR ("purpura, schoenlein henoch"[MeSH Terms] OR ("purpura"[All Fields] AND "schoenlein henoch"[All Fields]) OR "schoenlein-henoch purpura"[All Fields] OR ("anaphylactoid"[All Fields] AND "purpura"[All Fields]) OR "anaphylactoid purpura"[All Fields]) OR (("anaphylactic"[All Fields] OR "anaphylactically"[All Fields]) AND ("purpura"[MeSH Terms] OR "purpura"[All Fields] OR "purpuras"[All Fields])) | 6361 |
| #2 | (("gastrointestinal"[All Fields] OR "gastrointestinally"[All Fields] OR "gastrointestine"[All Fields]) AND "symptom*"[All Fields]) OR (("gastrointestinal"[All Fields] OR "gastrointestinally"[All Fields] OR "gastrointestine"[All Fields]) AND ("manifest"[All Fields] OR "manifestating"[All Fields] OR "manifestation"[All Fields] OR "manifestations"[All Fields] OR "manifested"[All Fields] OR "manifesting"[All Fields] OR "manifestion"[All Fields] OR "manifestions"[All Fields] OR "manifests"[All Fields])) OR (("gastrointestinal"[All Fields] OR "gastrointestinally"[All Fields] OR "gastrointestine"[All Fields]) AND ("involve"[All Fields] OR "involved"[All Fields] OR "involvement"[All Fields] OR "involvements"[All Fields] OR "involves"[All Fields] OR "involving"[All Fields] OR "involvment"[All Fields])) OR (("gastrointestinal"[All Fields] OR "gastrointestinally"[All Fields] OR "gastrointestine"[All Fields]) AND ("pain"[MeSH Terms] OR "pain"[All Fields])) OR (("abdomen"[MeSH Terms] OR "abdomen"[All Fields] OR "abdominal"[All Fields] OR "abdominally"[All Fields] OR "abdominals"[All Fields]) AND "symptom*"[All Fields]) OR (("abdomen"[MeSH Terms] OR "abdomen"[All Fields] OR "abdominal"[All Fields] OR "abdominally"[All Fields] OR "abdominals"[All Fields]) AND ("manifest"[All Fields] OR "manifestating"[All Fields] OR "manifestation"[All Fields] OR "manifestations"[All Fields] OR "manifested"[All Fields] OR "manifesting"[All Fields] OR "manifestion"[All Fields] OR "manifestions"[All Fields] OR "manifests"[All Fields])) OR (("abdomen"[MeSH Terms] OR "abdomen"[All Fields] OR "abdominal"[All Fields] OR "abdominally"[All Fields] OR "abdominals"[All Fields]) AND ("involve"[All Fields] OR "involved"[All Fields] OR "involvement"[All Fields] OR "involvements"[All Fields] OR "involves"[All Fields] OR "involving"[All Fields] OR "involvment"[All Fields])) OR ("abdominal pain"[MeSH Terms] OR ("abdominal"[All Fields] AND "pain"[All Fields]) OR "abdominal pain"[All Fields]) OR ("stomachache"[All Fields] OR "stomachaches"[All Fields]) OR ("colic"[MeSH Terms] OR "colic"[All Fields] OR "colics"[All Fields]) OR ("vomiter"[All Fields] OR "vomiters"[All Fields] OR "vomiting"[MeSH Terms] OR "vomiting"[All Fields] OR "vomit"[All Fields] OR "vomited"[All Fields] OR "vomits"[All Fields] OR "vomitings"[All Fields] OR "vomition"[All Fields] OR "vomitting"[All Fields]) OR ("gastrointestinal hemorrhage"[MeSH Terms] OR ("gastrointestinal"[All Fields] AND "hemorrhage"[All Fields]) OR "gastrointestinal hemorrhage"[All Fields] OR ("bloody"[All Fields] AND "stool"[All Fields]) OR "bloody stool"[All Fields]) OR ("perforant"[All Fields] OR "perforants"[All Fields] OR "perforate"[All Fields] OR "perforated"[All Fields] OR "perforates"[All Fields] OR "perforating"[All Fields] OR "perforation"[All Fields] OR "perforations"[All Fields] OR "perforative"[All Fields] OR "perforator"[All Fields] OR "perforator s"[All Fields] OR "perforators"[All Fields]) OR ("gastrointestinal hemorrhage"[MeSH Terms] OR ("gastrointestinal"[All Fields] AND "hemorrhage"[All Fields]) OR "gastrointestinal hemorrhage"[All Fields] OR ("gastrointestinal"[All Fields] AND "bleeding"[All Fields]) OR "gastrointestinal bleeding"[All Fields]) OR (("abdomen"[MeSH Terms] OR "abdomen"[All Fields] OR "abdominal"[All Fields] OR "abdominally"[All Fields] OR "abdominals"[All Fields]) AND ("bleedings"[All Fields] OR "hemorrhage"[MeSH Terms] OR "hemorrhage"[All Fields] OR "bleed"[All Fields] OR "bleeding"[All Fields] OR "bleeds"[All Fields])) OR ("gastritis"[MeSH Terms] OR "gastritis"[All Fields] OR "gastritides"[All Fields]) OR ("duodenitis"[MeSH Terms] OR "duodenitis"[All Fields] OR "duodenum"[MeSH Terms] OR "duodenum"[All Fields] OR "duodenal"[All Fields]) OR (("periumbilical"[All Fields] OR "periumbilically"[All Fields]) AND ("pain"[MeSH Terms] OR "pain"[All Fields])) OR (("epigastric"[All Fields] OR "epigastrical"[All Fields]) AND ("pain"[MeSH Terms] OR "pain"[All Fields])) OR ("intussuscepted"[All Fields] OR "intussuscepting"[All Fields] OR "intussusception"[MeSH Terms] OR "intussusception"[All Fields] OR "intussusceptions"[All Fields]) OR ("nausea"[MeSH Terms] OR "nausea"[All Fields] OR "nauseas"[All Fields]) OR ("ulcer"[MeSH Terms] OR "ulcer"[All Fields] OR "ulcerate"[All Fields] OR "ulcerated"[All Fields] OR "ulcerates"[All Fields] OR "ulcerating"[All Fields] OR "ulceration"[All Fields] OR "ulcerations"[All Fields] OR "ulcerative"[All Fields] OR "ulcers"[All Fields] OR "ulcer s"[All Fields] OR "ulcerous"[All Fields]) | 1048522 |
| #3 | signs and symptoms, digestive[MeSH Terms] OR "gastrointestinal diseases"[MeSH Terms] | 1030374 |
| #4 | #1 AND (#2 OR #3) | 1637 |
| #5 | "proton pump inhibitors"[Pharmacological Action] OR "proton pump inhibitors"[MeSH Terms] OR ("proton"[All Fields] AND "pump"[All Fields] AND "inhibitors"[All Fields]) OR "proton pump inhibitors"[All Fields] OR ("proton"[All Fields] AND "pump"[All Fields] AND "inhibitor"[All Fields]) OR "proton pump inhibitor"[All Fields] | 27724 |
| #6 | "histamine h2 antagonists"[Pharmacological Action] OR "histamine h2 antagonists"[MeSH Terms] OR ("histamine"[All Fields] AND "h2"[All Fields] AND "antagonists"[All Fields]) OR "histamine h2 antagonists"[All Fields] OR ("h2"[All Fields] AND "blocker"[All Fields]) OR "h2 blocker"[All Fields] | 21917 |
| #7 | "anti ulcer agents"[Pharmacological Action] OR "anti ulcer agents"[MeSH Terms] OR ("anti ulcer"[All Fields] AND "agents"[All Fields]) OR "anti ulcer agents"[All Fields] OR ("anti"[All Fields] AND "ulcer"[All Fields] AND "drug"[All Fields]) OR "anti ulcer drug"[All Fields] | 60580 |
| #8 | #4 AND (#5 OR #6 OR #7) | 19 |
| #9 | "prevent"[All Fields] OR "preventability"[All Fields] OR "preventable"[All Fields] OR "preventative"[All Fields] OR "preventatively"[All Fields] OR "preventatives"[All Fields] OR "prevented"[All Fields] OR "preventing"[All Fields] OR "prevention and control"[MeSH Subheading] OR ("prevention"[All Fields] AND "control"[All Fields]) OR "prevention and control"[All Fields] OR "prevention"[All Fields] OR "prevention s"[All Fields] OR "preventions"[All Fields] OR "preventive"[All Fields] OR "preventively"[All Fields] OR "preventives"[All Fields] OR "prevents"[All Fields] | 2762962 |
| #10 | #4 AND #9 | 85 |
| #11 | #8 OR #10 | 102 |
| 12 | #11 AND (Meta-Analysis［PT］OR systematic［SB］） | 4 |
| 13 | #11 AND "Randomized Controlled Trial"［PT］ | 6 |

**CQ7: What additional treatments are available for pediatric patients with severe/refractory IgA vasculitis who are not adequately responsive to glucocorticoids (excluding nephritis)?**

**7-1. Is steroid pulse therapy recommended for pediatric patients with severe/refractory IgA vasculitis who are not adequately responsive to glucocorticoids (excluding nephritis)?**

Search date：27-Oct-2021

| No. | Search expression | Number of searches |
| --- | --- | --- |
| #1 | "purpura, schoenlein henoch"[MeSH Terms] OR ("purpura"[All Fields] AND "schoenlein henoch"[All Fields]) OR "schoenlein-henoch purpura"[All Fields] OR ("purpura"[All Fields] AND "schoenlein"[All Fields] AND "henoch"[All Fields]) OR "purpura schoenlein henoch"[All Fields] OR ("purpura, schoenlein henoch"[MeSH Terms] OR ("purpura"[All Fields] AND "schoenlein henoch"[All Fields]) OR "schoenlein-henoch purpura"[All Fields] OR ("purpura"[All Fields] AND "schonlein"[All Fields] AND "henoch"[All Fields]) OR "purpura schonlein henoch"[All Fields]) OR ("purpura, schoenlein henoch"[MeSH Terms] OR ("purpura"[All Fields] AND "schoenlein henoch"[All Fields]) OR "schoenlein-henoch purpura"[All Fields] OR "immunoglobulin a vasculitis"[All Fields]) OR ("IgA"[All Fields] AND ("vasculitide"[All Fields] OR "vasculities"[All Fields] OR "vasculitis"[MeSH Terms] OR "vasculitis"[All Fields] OR "vasculitides"[All Fields])) OR ("purpura, schoenlein henoch"[MeSH Terms] OR ("purpura"[All Fields] AND "schoenlein henoch"[All Fields]) OR "schoenlein-henoch purpura"[All Fields] OR ("allergic"[All Fields] AND "purpura"[All Fields]) OR "allergic purpura"[All Fields]) OR ("purpura, schoenlein henoch"[MeSH Terms] OR ("purpura"[All Fields] AND "schoenlein henoch"[All Fields]) OR "schoenlein-henoch purpura"[All Fields] OR ("anaphylactoid"[All Fields] AND "purpura"[All Fields]) OR "anaphylactoid purpura"[All Fields]) OR (("anaphylactic"[All Fields] OR "anaphylactically"[All Fields]) AND ("purpura"[MeSH Terms] OR "purpura"[All Fields] OR "purpuras"[All Fields])) | 6,346 |
| #2 | "steroidal"[All Fields] OR "steroidals"[All Fields] OR "steroidic"[All Fields] OR "steroids"[MeSH Terms] OR "steroids"[All Fields] OR "steroid"[All Fields] OR ("glucocorticoids"[Pharmacological Action] OR "glucocorticoids"[MeSH Terms] OR "glucocorticoids"[All Fields] OR "glucocorticoid"[All Fields]) OR ("adrenal cortex hormones"[MeSH Terms] OR ("adrenal"[All Fields] AND "cortex"[All Fields] AND "hormones"[All Fields]) OR "adrenal cortex hormones"[All Fields] OR "corticosteroid"[All Fields] OR "corticosteroids"[All Fields] OR "corticosteroidal"[All Fields] OR "corticosteroide"[All Fields] OR "corticosteroides"[All Fields]) OR ("hydrocortisone"[MeSH Terms] OR "hydrocortisone"[All Fields] OR "hydrocortisones"[All Fields]) OR ("dexamethason"[All Fields] OR "dexamethasone"[MeSH Terms] OR "dexamethasone"[All Fields] OR "dexamethasone s"[All Fields] OR "dexamethasones"[All Fields]) OR ("prednison"[All Fields] OR "prednisone"[MeSH Terms] OR "prednisone"[All Fields]) OR ("prednisolon"[All Fields] OR "prednisolone"[MeSH Terms] OR "prednisolone"[All Fields]) OR ("methylprednisolone"[MeSH Terms] OR "methylprednisolone"[All Fields] OR "methylprednisolon"[All Fields]) OR ("adrenal cortex hormones"[MeSH Terms] OR ("adrenal"[All Fields] AND "cortex"[All Fields] AND "hormones"[All Fields]) OR "adrenal cortex hormones"[All Fields] OR ("adrenal"[All Fields] AND "cortex"[All Fields] AND "hormone"[All Fields]) OR "adrenal cortex hormone"[All Fields]) | 1,197,835 |
| #3 | ("steroidal"[All Fields] OR "steroidals"[All Fields] OR "steroidic"[All Fields] OR "steroids"[MeSH Terms] OR "steroids"[All Fields] OR "steroid"[All Fields] OR ("glucocorticoids"[Pharmacological Action] OR "glucocorticoids"[MeSH Terms] OR "glucocorticoids"[All Fields] OR "glucocorticoid"[All Fields]) OR ("adrenal cortex hormones"[MeSH Terms] OR ("adrenal"[All Fields] AND "cortex"[All Fields] AND "hormones"[All Fields]) OR "adrenal cortex hormones"[All Fields] OR "corticosteroid"[All Fields] OR "corticosteroids"[All Fields] OR "corticosteroidal"[All Fields] OR "corticosteroide"[All Fields] OR "corticosteroides"[All Fields]) OR ("hydrocortisone"[MeSH Terms] OR "hydrocortisone"[All Fields] OR "hydrocortisones"[All Fields]) OR ("dexamethason"[All Fields] OR "dexamethasone"[MeSH Terms] OR "dexamethasone"[All Fields] OR "dexamethasone s"[All Fields] OR "dexamethasones"[All Fields]) OR ("prednison"[All Fields] OR "prednisone"[MeSH Terms] OR "prednisone"[All Fields]) OR ("prednisolon"[All Fields] OR "prednisolone"[MeSH Terms] OR "prednisolone"[All Fields]) OR ("methylprednisolone"[MeSH Terms] OR "methylprednisolone"[All Fields] OR "methylprednisolon"[All Fields]) OR ("adrenal cortex hormones"[MeSH Terms] OR ("adrenal"[All Fields] AND "cortex"[All Fields] AND "hormones"[All Fields]) OR "adrenal cortex hormones"[All Fields] OR ("adrenal"[All Fields] AND "cortex"[All Fields] AND "hormone"[All Fields]) OR "adrenal cortex hormone"[All Fields])) AND "pulse*"[All Fields] | 16,898 |
| #4 | "methylprednisolone"[MeSH Terms] OR "methylprednisolone"[All Fields] OR "methylprednisolon"[All Fields] | 27,932 |
| #5 | ("steroidal"[All Fields] OR "steroidals"[All Fields] OR "steroidic"[All Fields] OR "steroids"[MeSH Terms] OR "steroids"[All Fields] OR "steroid"[All Fields]) AND "pulse*"[All Fields] | 13,952 |
| #6 | pulse therapy*[All Fields] OR "pulse therapy, drug"[MeSH Terms] | 142,245 |
| #7 | "high"[All Fields] AND "dose"[All Fields] AND ("steroidal"[All Fields] OR "steroidals"[All Fields] OR "steroidic"[All Fields] OR "steroids"[MeSH Terms] OR "steroids"[All Fields] OR "steroid"[All Fields]) | 34,640 |
| #8 | #1 AND (#3 OR #4 OR #5 OR #6 OR #7 | 280 |

**7-2. Is intravenous immunoglobulin recommended for pediatric patients with severe/refractory IgA vasculitis who are not adequately responsive to glucocorticoids (excluding nephritis)?**

Search date：27-Oct-2021

| No. | Search expression | Number of searches |
| --- | --- | --- |
| #1 | "purpura, schoenlein henoch"[MeSH Terms] OR ("purpura"[All Fields] AND "schoenlein henoch"[All Fields]) OR "schoenlein-henoch purpura"[All Fields] OR ("purpura"[All Fields] AND "schoenlein"[All Fields] AND "henoch"[All Fields]) OR "purpura schoenlein henoch"[All Fields] OR ("purpura, schoenlein henoch"[MeSH Terms] OR ("purpura"[All Fields] AND "schoenlein henoch"[All Fields]) OR "schoenlein-henoch purpura"[All Fields] OR ("purpura"[All Fields] AND "schonlein"[All Fields] AND "henoch"[All Fields]) OR "purpura schonlein henoch"[All Fields]) OR ("purpura, schoenlein henoch"[MeSH Terms] OR ("purpura"[All Fields] AND "schoenlein henoch"[All Fields]) OR "schoenlein-henoch purpura"[All Fields] OR "immunoglobulin a vasculitis"[All Fields]) OR ("IgA"[All Fields] AND ("vasculitide"[All Fields] OR "vasculities"[All Fields] OR "vasculitis"[MeSH Terms] OR "vasculitis"[All Fields] OR "vasculitides"[All Fields])) OR ("purpura, schoenlein henoch"[MeSH Terms] OR ("purpura"[All Fields] AND "schoenlein henoch"[All Fields]) OR "schoenlein-henoch purpura"[All Fields] OR ("allergic"[All Fields] AND "purpura"[All Fields]) OR "allergic purpura"[All Fields]) OR ("purpura, schoenlein henoch"[MeSH Terms] OR ("purpura"[All Fields] AND "schoenlein henoch"[All Fields]) OR "schoenlein-henoch purpura"[All Fields] OR ("anaphylactoid"[All Fields] AND "purpura"[All Fields]) OR "anaphylactoid purpura"[All Fields]) OR (("anaphylactic"[All Fields] OR "anaphylactically"[All Fields]) AND ("purpura"[MeSH Terms] OR "purpura"[All Fields] OR "purpuras"[All Fields])) | 6346 |
| #2 | immunoglobulins, intravenous[MeSH Terms] OR ("immunoglobulins"[All Fields] AND "intravenous"[All Fields]) OR "intravenous immunoglobulins"[All Fields] OR ("intravenous"[All Fields] AND "immunoglobulin"[All Fields]) OR "intravenous immunoglobulin"[All Fields] | 27516 |
| #3 | immunoglobulins, intravenous[MeSH Terms] OR ("immunoglobulins"[All Fields] AND "intravenous"[All Fields]) OR "intravenous immunoglobulins"[All Fields] OR "ivig"[All Fields] | 20185 |
| #4 | #1 AND (#2 OR #3) | 134 |

**7-3. Are immunosuppressants recommended for pediatric patients with severe/refractory IgA vasculitis who are not adequately responsive to glucocorticoids (excluding nephritis)?**

Search date：9-Nov-2021

| No. | Search expression | Number of searches |
| --- | --- | --- |
| #1 | "purpura, schoenlein henoch"[MeSH Terms] OR ("purpura"[All Fields] AND "schoenlein henoch"[All Fields]) OR "schoenlein-henoch purpura"[All Fields] OR ("purpura"[All Fields] AND "schoenlein"[All Fields] AND "henoch"[All Fields]) OR "purpura schoenlein henoch"[All Fields] OR ("purpura, schoenlein henoch"[MeSH Terms] OR ("purpura"[All Fields] AND "schoenlein henoch"[All Fields]) OR "schoenlein-henoch purpura"[All Fields] OR ("purpura"[All Fields] AND "schonlein"[All Fields] AND "henoch"[All Fields]) OR "purpura schonlein henoch"[All Fields]) OR ("purpura, schoenlein henoch"[MeSH Terms] OR ("purpura"[All Fields] AND "schoenlein henoch"[All Fields]) OR "schoenlein-henoch purpura"[All Fields] OR "immunoglobulin a vasculitis"[All Fields]) OR ("IgA"[All Fields] AND ("vasculitide"[All Fields] OR "vasculities"[All Fields] OR "vasculitis"[MeSH Terms] OR "vasculitis"[All Fields] OR "vasculitides"[All Fields])) OR ("purpura, schoenlein henoch"[MeSH Terms] OR ("purpura"[All Fields] AND "schoenlein henoch"[All Fields]) OR "schoenlein-henoch purpura"[All Fields] OR ("allergic"[All Fields] AND "purpura"[All Fields]) OR "allergic purpura"[All Fields]) OR ("purpura, schoenlein henoch"[MeSH Terms] OR ("purpura"[All Fields] AND "schoenlein henoch"[All Fields]) OR "schoenlein-henoch purpura"[All Fields] OR ("anaphylactoid"[All Fields] AND "purpura"[All Fields]) OR "anaphylactoid purpura"[All Fields]) OR (("anaphylactic"[All Fields] OR "anaphylactically"[All Fields]) AND ("purpura"[MeSH Terms] OR "purpura"[All Fields] OR "purpuras"[All Fields])) | 4,935 |
| #2 | immunosuppressive agents[Pharmacological Action] OR "immunosuppressive agents"[MeSH Terms] OR ("immunosuppressive"[All Fields] AND "agents"[All Fields]) OR "immunosuppressive agents"[All Fields] OR ("immunosuppressive"[All Fields] AND "agent"[All Fields]) OR "immunosuppressive agent"[All Fields] OR ("immunosuppressive agents"[Pharmacological Action] OR "immunosuppressive agents"[MeSH Terms] OR ("immunosuppressive"[All Fields] AND "agents"[All Fields]) OR "immunosuppressive agents"[All Fields] OR ("immunosuppressant"[All Fields] AND "agent"[All Fields]) OR "immunosuppressant agent"[All Fields]) OR "immunosuppressant*"[All Fields] | 329,926 |
| #3 | "azathioprin"[All Fields] OR "azathioprine"[MeSH Terms] OR "azathioprine"[All Fields] OR ("cyclophosphamide"[MeSH Terms] OR "cyclophosphamide"[All Fields] OR "cyclophosphamid"[All Fields] OR "cyclophosphamide s"[All Fields] OR "cyclophosphamides"[All Fields]) OR ("cyclosporine"[MeSH Terms] OR "cyclosporine"[All Fields] OR "ciclosporin"[All Fields] OR "ciclosporine"[All Fields] OR "cyclosporin"[All Fields] OR "cyclosporine s"[All Fields] OR "cyclosporins"[MeSH Terms] OR "cyclosporins"[All Fields] OR "cyclosporines"[All Fields]) OR ("tacrolimus"[MeSH Terms] OR "tacrolimus"[All Fields]) OR ("tacrolimus"[MeSH Terms] OR "tacrolimus"[All Fields] OR "fk506"[All Fields]) OR ("tacrolimus"[MeSH Terms] OR "tacrolimus"[All Fields] OR "fk 506"[All Fields]) OR ("mizoribin"[All Fields] OR "mizoribine"[Supplementary Concept] OR "mizoribine"[All Fields]) OR ("mizoribine"[Supplementary Concept] OR "mizoribine"[All Fields] OR "bredinin"[All Fields]) OR ("mycophenolic acid"[MeSH Terms] OR ("mycophenolic"[All Fields] AND "acid"[All Fields]) OR "mycophenolic acid"[All Fields] OR ("mycophenolate"[All Fields] AND "mofetil"[All Fields]) OR "mycophenolate mofetil"[All Fields]) OR "mycophenol*"[All Fields] OR ("mycophenolic acid"[MeSH Terms] OR ("mycophenolic"[All Fields] AND "acid"[All Fields]) OR "mycophenolic acid"[All Fields] OR "cellcept"[All Fields] OR ("mycophenolate"[All Fields] AND "mofetil"[All Fields]) OR "mycophenolate mofetil"[All Fields]) OR ("rituximab"[MeSH Terms] OR "rituximab"[All Fields] OR "rituximab s"[All Fields]) OR ("rituximab"[MeSH Terms] OR "rituximab"[All Fields] OR "rituxan"[All Fields] OR "rituximab s"[All Fields]) | 170,408 |
| #4 | #1 AND (#2 OR #3) | 490 |
| #5 | "paediatrics"[All Fields] OR "pediatrics"[MeSH Terms] OR "pediatrics"[All Fields] OR "paediatric"[All Fields] OR "pediatric"[All Fields] OR "child"[MeSH Terms] OR "child"[All Fields] OR "children"[All Fields] OR "child s"[All Fields] OR "children s"[All Fields] OR "childrens"[All Fields] OR "childs"[All Fields] OR "infant"[MeSH Terms] OR "infant"[All Fields] OR "infants"[All Fields] OR "infant s"[All Fields] OR "childhood"[All Fields] OR "childhoods"[All Fields] | 3,019,773 |
| #6 | #4 AND #5 | 253 |

(#6 only children)

**7-4. Is plasma exchange recommended for pediatric patients with severe/refractory IgA vasculitis who are not adequately responsive to glucocorticoids (excluding nephritis)?**

Search date：1-Dec-2021

| No. | Search expression | Number of searches |
| --- | --- | --- |
| #1 | "purpura, schoenlein henoch"[MeSH Terms] OR ("purpura"[All Fields] AND "schoenlein henoch"[All Fields]) OR "schoenlein-henoch purpura"[All Fields] OR ("purpura"[All Fields] AND "schoenlein"[All Fields] AND "henoch"[All Fields]) OR "purpura schoenlein henoch"[All Fields] OR ("purpura, schoenlein henoch"[MeSH Terms] OR ("purpura"[All Fields] AND "schoenlein henoch"[All Fields]) OR "schoenlein-henoch purpura"[All Fields] OR ("purpura"[All Fields] AND "schonlein"[All Fields] AND "henoch"[All Fields]) OR "purpura schonlein henoch"[All Fields]) OR ("purpura, schoenlein henoch"[MeSH Terms] OR ("purpura"[All Fields] AND "schoenlein henoch"[All Fields]) OR "schoenlein-henoch purpura"[All Fields] OR "immunoglobulin a vasculitis"[All Fields]) OR ("IgA"[All Fields] AND ("vasculitide"[All Fields] OR "vasculities"[All Fields] OR "vasculitis"[MeSH Terms] OR "vasculitis"[All Fields] OR "vasculitides"[All Fields])) OR ("purpura, schoenlein henoch"[MeSH Terms] OR ("purpura"[All Fields] AND "schoenlein henoch"[All Fields]) OR "schoenlein-henoch purpura"[All Fields] OR ("allergic"[All Fields] AND "purpura"[All Fields]) OR "allergic purpura"[All Fields]) OR ("purpura, schoenlein henoch"[MeSH Terms] OR ("purpura"[All Fields] AND "schoenlein henoch"[All Fields]) OR "schoenlein-henoch purpura"[All Fields] OR ("anaphylactoid"[All Fields] AND "purpura"[All Fields]) OR "anaphylactoid purpura"[All Fields]) OR (("anaphylactic"[All Fields] OR "anaphylactically"[All Fields]) AND ("purpura"[MeSH Terms] OR "purpura"[All Fields] OR "purpuras"[All Fields])) | 6097 |
| #2 | "plasmapheresis"[MeSH Terms] OR "plasmapheresis"[All Fields] OR ("plasma"[All Fields] AND "exchange"[All Fields]) OR "plasma exchange"[All Fields] OR "plasma exchange"[MeSH Terms] OR ("plasma"[All Fields] AND "exchange"[All Fields]) OR ("blood component removal"[MeSH Terms] OR ("blood"[All Fields] AND "component"[All Fields] AND "removal"[All Fields]) OR "blood component removal"[All Fields] OR "apheresis"[All Fields]) OR (("plasma"[MeSH Terms] OR "plasma"[All Fields] OR "plasmas"[All Fields] OR "plasma s"[All Fields]) AND ("infusate"[All Fields] OR "infusates"[All Fields] OR "infuse"[All Fields] OR "infused"[All Fields] OR "infuser"[All Fields] OR "infusers"[All Fields] OR "infuses"[All Fields] OR "infusing"[All Fields] OR "infusion"[All Fields] OR "infusions"[All Fields])) OR "plasmapharesis"[All Fields] | 107,496 |
| #3 | #1 AND #2) | 196 |

**CQ8: Are glucocorticoids recommended for the prevention of pediatric IgA vasculitis nephritis?**

Search date：10-Nov-2021

| No. | Search expression | Number of searches |
| --- | --- | --- |
| #1 | "purpura, schoenlein henoch"[MeSH Terms] OR ("purpura"[All Fields] AND "schoenlein henoch"[All Fields]) OR "schoenlein-henoch purpura"[All Fields] OR ("purpura"[All Fields] AND "schoenlein"[All Fields] AND "henoch"[All Fields]) OR "purpura schoenlein henoch"[All Fields] OR ("purpura, schoenlein henoch"[MeSH Terms] OR ("purpura"[All Fields] AND "schoenlein henoch"[All Fields]) OR "schoenlein-henoch purpura"[All Fields] OR ("purpura"[All Fields] AND "schonlein"[All Fields] AND "henoch"[All Fields]) OR "purpura schonlein henoch"[All Fields]) OR ("purpura, schoenlein henoch"[MeSH Terms] OR ("purpura"[All Fields] AND "schoenlein henoch"[All Fields]) OR "schoenlein-henoch purpura"[All Fields] OR "immunoglobulin a vasculitis"[All Fields]) OR ("IgA"[All Fields] AND ("vasculitide"[All Fields] OR "vasculities"[All Fields] OR "vasculitis"[MeSH Terms] OR "vasculitis"[All Fields] OR "vasculitides"[All Fields])) OR ("purpura, schoenlein henoch"[MeSH Terms] OR ("purpura"[All Fields] AND "schoenlein henoch"[All Fields]) OR "schoenlein-henoch purpura"[All Fields] OR ("allergic"[All Fields] AND "purpura"[All Fields]) OR "allergic purpura"[All Fields]) OR ("purpura, schoenlein henoch"[MeSH Terms] OR ("purpura"[All Fields] AND "schoenlein henoch"[All Fields]) OR "schoenlein-henoch purpura"[All Fields] OR ("anaphylactoid"[All Fields] AND "purpura"[All Fields]) OR "anaphylactoid purpura"[All Fields]) OR (("anaphylactic"[All Fields] OR "anaphylactically"[All Fields]) AND ("purpura"[MeSH Terms] OR "purpura"[All Fields] OR "purpuras"[All Fields])) | 7128 |
| #2 | ("purpura"[MeSH Terms] OR "purpura"[All Fields] OR "purpuras"[All Fields]) AND ("nephritis"[MeSH Terms] OR "nephritis"[All Fields] OR "nephritides"[All Fields]) | 2178 |
| #3 | "steroidal"[All Fields] OR "steroidals"[All Fields] OR "steroidic"[All Fields] OR "steroids"[MeSH Terms] OR "steroids"[All Fields] OR "steroid"[All Fields] OR ("glucocorticoids"[Pharmacological Action] OR "glucocorticoids"[MeSH Terms] OR "glucocorticoids"[All Fields] OR "glucocorticoid"[All Fields]) OR ("adrenal cortex hormones"[MeSH Terms] OR ("adrenal"[All Fields] AND "cortex"[All Fields] AND "hormones"[All Fields]) OR "adrenal cortex hormones"[All Fields] OR "corticosteroid"[All Fields] OR "corticosteroids"[All Fields] OR "corticosteroidal"[All Fields] OR "corticosteroide"[All Fields] OR "corticosteroides"[All Fields]) OR ("hydrocortisone"[MeSH Terms] OR "hydrocortisone"[All Fields] OR "hydrocortisones"[All Fields]) OR ("dexamethason"[All Fields] OR "dexamethasone"[MeSH Terms] OR "dexamethasone"[All Fields] OR "dexamethasone s"[All Fields] OR "dexamethasones"[All Fields]) OR ("prednison"[All Fields] OR "prednisone"[MeSH Terms] OR "prednisone"[All Fields]) OR ("prednisolon"[All Fields] OR "prednisolone"[MeSH Terms] OR "prednisolone"[All Fields]) OR ("methylprednisolone"[MeSH Terms] OR "methylprednisolone"[All Fields] OR "methylprednisolon"[All Fields]) OR ("adrenal cortex hormones"[MeSH Terms] OR ("adrenal"[All Fields] AND "cortex"[All Fields] AND "hormones"[All Fields]) OR "adrenal cortex hormones"[All Fields] OR ("adrenal"[All Fields] AND "cortex"[All Fields] AND "hormone"[All Fields]) OR "adrenal cortex hormone"[All Fields]) | 1270608 |
| #4 | "prevent*"[All Fields] | 2817212 |
| #5 | prevention and control[MeSH Subheading] OR ("prevention"[All Fields] AND "control"[All Fields]) OR "prevention and control"[All Fields] OR "prophylaxis"[All Fields] OR "prophylaxies"[All Fields] OR "prophylaxy"[All Fields] | 1603440 |
| #6 | (#1 OR #2) AND #3 AND (#4 OR #5) | 115 |

**CQ9: Are renin–angiotensin system (RA system) inhibitors recommended for pediatric IgA vasculitis nephritis?**

Search date：21-Nov-2021

| No. | Search expression | Number of searches |
| --- | --- | --- |
| #1 | "purpura, schoenlein henoch"[MeSH Terms] OR ("purpura"[All Fields] AND "schoenlein henoch"[All Fields]) OR "schoenlein-henoch purpura"[All Fields] OR ("purpura"[All Fields] AND "schoenlein"[All Fields] AND "henoch"[All Fields]) OR "purpura schoenlein henoch"[All Fields] OR ("purpura, schoenlein henoch"[MeSH Terms] OR ("purpura"[All Fields] AND "schoenlein henoch"[All Fields]) OR "schoenlein-henoch purpura"[All Fields] OR ("purpura"[All Fields] AND "schonlein"[All Fields] AND "henoch"[All Fields]) OR "purpura schonlein henoch"[All Fields]) OR ("purpura, schoenlein henoch"[MeSH Terms] OR ("purpura"[All Fields] AND "schoenlein henoch"[All Fields]) OR "schoenlein-henoch purpura"[All Fields] OR "immunoglobulin a vasculitis"[All Fields]) OR ("IgA"[All Fields] AND ("vasculitide"[All Fields] OR "vasculities"[All Fields] OR "vasculitis"[MeSH Terms] OR "vasculitis"[All Fields] OR "vasculitides"[All Fields])) OR ("purpura, schoenlein henoch"[MeSH Terms] OR ("purpura"[All Fields] AND "schoenlein henoch"[All Fields]) OR "schoenlein-henoch purpura"[All Fields] OR ("allergic"[All Fields] AND "purpura"[All Fields]) OR "allergic purpura"[All Fields]) OR ("purpura, schoenlein henoch"[MeSH Terms] OR ("purpura"[All Fields] AND "schoenlein henoch"[All Fields]) OR "schoenlein-henoch purpura"[All Fields] OR ("anaphylactoid"[All Fields] AND "purpura"[All Fields]) OR "anaphylactoid purpura"[All Fields]) OR (("anaphylactic"[All Fields] OR "anaphylactically"[All Fields]) AND ("purpura"[MeSH Terms] OR "purpura"[All Fields] OR "purpuras"[All Fields])) | 7,100 |
| #2 | ("purpura"[MeSH Terms] OR "purpura"[All Fields] OR "purpuras"[All Fields]) AND ("nephritis"[MeSH Terms] OR "nephritis"[All Fields] OR "nephritides"[All Fields]) | 2,165 |
| #3 | "ACE-I"[All Fields] | 2,452 |
| #4 | "angiotensin converting enzyme inhibitors"[Pharmacological Action] OR "angiotensin converting enzyme inhibitors"[MeSH Terms] OR ("angiotensin converting"[All Fields] AND "enzyme"[All Fields] AND "inhibitors"[All Fields]) OR "angiotensin converting enzyme inhibitors"[All Fields] OR ("angiotensin"[All Fields] AND "converting"[All Fields] AND "enzyme"[All Fields] AND "inhibitor"[All Fields]) OR "angiotensin converting enzyme inhibitor"[All Fields] | 58,656 |
| #5 | "angiotensin converting enzyme inhibitors"[Pharmacological Action] OR "angiotensin converting enzyme inhibitors"[MeSH Terms] OR ("angiotensin converting"[All Fields] AND "enzyme"[All Fields] AND "inhibitors"[All Fields]) OR "angiotensin converting enzyme inhibitors"[All Fields] OR ("ace"[All Fields] AND "inhibitor"[All Fields]) OR "ace inhibitor"[All Fields] | 57,518 |
| #6 | "ARB"[All Fields] | 7,001 |
| #07 | "angiotensin receptor antagonists"[Pharmacological Action] OR "angiotensin receptor antagonists"[MeSH Terms] OR ("angiotensin"[All Fields] AND "receptor"[All Fields] AND "antagonists"[All Fields]) OR "angiotensin receptor antagonists"[All Fields] OR ("angiotensin"[All Fields] AND "receptor"[All Fields] AND "blocker"[All Fields]) OR "angiotensin receptor blocker"[All Fields] | 36,236 |
| #8 | ("renin angiotensin system"[MeSH Terms] OR ("renin angiotensin"[All Fields] AND "system"[All Fields]) OR "renin angiotensin system"[All Fields] OR ("renin"[All Fields] AND "angiotensin"[All Fields] AND "system"[All Fields]) OR "renin angiotensin system"[All Fields]) AND ("blocker"[All Fields] OR "blocker s"[All Fields] OR "blockers"[All Fields]) | 7,517 |
| #9 | ("renin angiotensin system"[MeSH Terms] OR ("renin angiotensin"[All Fields] AND "system"[All Fields]) OR "renin angiotensin system"[All Fields] OR ("renin"[All Fields] AND "angiotensin"[All Fields] AND "aldosterone"[All Fields] AND "system"[All Fields]) OR "renin angiotensin aldosterone system"[All Fields]) AND ("antagonists and inhibitors"[MeSH Subheading] OR ("antagonists"[All Fields] AND "inhibitors"[All Fields]) OR "antagonists and inhibitors"[All Fields] OR "inhibitors"[All Fields] OR "inhibitor"[All Fields] OR "inhibitor s"[All Fields]) | 13,776 |
| #10 | (#1 OR #2) AND (#3 OR #4 OR #5 OR #6 OR #7 OR #8 OR #9) | 62 |

**CQ10: Are glucocorticoids and immunosuppressants recommended for severe cases of pediatric IgA vasculitis nephritis?**

**10-1. Are steroid and immunosuppressants recommended for severe cases of pediatric IgA vasculitis nephritis?**

Search date：31-Oct-2021

| No. | Search expression | Number of searches |
| --- | --- | --- |
| #1 | "purpura, schoenlein henoch"[MeSH Terms] OR ("purpura"[All Fields] AND "schoenlein henoch"[All Fields]) OR "schoenlein-henoch purpura"[All Fields] OR ("purpura"[All Fields] AND "schoenlein"[All Fields] AND "henoch"[All Fields]) OR "purpura schoenlein henoch"[All Fields] OR ("purpura, schoenlein henoch"[MeSH Terms] OR ("purpura"[All Fields] AND "schoenlein henoch"[All Fields]) OR "schoenlein-henoch purpura"[All Fields] OR ("purpura"[All Fields] AND "schonlein"[All Fields] AND "henoch"[All Fields]) OR "purpura schonlein henoch"[All Fields]) OR ("purpura, schoenlein henoch"[MeSH Terms] OR ("purpura"[All Fields] AND "schoenlein henoch"[All Fields]) OR "schoenlein-henoch purpura"[All Fields] OR "immunoglobulin a vasculitis"[All Fields]) OR ("IgA"[All Fields] AND ("vasculitide"[All Fields] OR "vasculities"[All Fields] OR "vasculitis"[MeSH Terms] OR "vasculitis"[All Fields] OR "vasculitides"[All Fields])) OR ("purpura, schoenlein henoch"[MeSH Terms] OR ("purpura"[All Fields] AND "schoenlein henoch"[All Fields]) OR "schoenlein-henoch purpura"[All Fields] OR ("allergic"[All Fields] AND "purpura"[All Fields]) OR "allergic purpura"[All Fields]) OR ("purpura, schoenlein henoch"[MeSH Terms] OR ("purpura"[All Fields] AND "schoenlein henoch"[All Fields]) OR "schoenlein-henoch purpura"[All Fields] OR ("anaphylactoid"[All Fields] AND "purpura"[All Fields]) OR "anaphylactoid purpura"[All Fields]) OR (("anaphylactic"[All Fields] OR "anaphylactically"[All Fields]) AND ("purpura"[MeSH Terms] OR "purpura"[All Fields] OR "purpuras"[All Fields])) | 7,100 |
| #2 | ("purpura"[MeSH Terms] OR "purpura"[All Fields] OR "purpuras"[All Fields]) AND ("nephritis"[MeSH Terms] OR "nephritis"[All Fields] OR "nephritides"[All Fields]) | 2,165 |
| #3 | "steroidal"[All Fields] OR "steroidals"[All Fields] OR "steroidic"[All Fields] OR "steroids"[MeSH Terms] OR "steroids"[All Fields] OR "steroid"[All Fields] OR ("glucocorticoids"[Pharmacological Action] OR "glucocorticoids"[MeSH Terms] OR "glucocorticoids"[All Fields] OR "glucocorticoid"[All Fields]) OR ("adrenal cortex hormones"[MeSH Terms] OR ("adrenal"[All Fields] AND "cortex"[All Fields] AND "hormones"[All Fields]) OR "adrenal cortex hormones"[All Fields] OR "corticosteroid"[All Fields] OR "corticosteroids"[All Fields] OR "corticosteroidal"[All Fields] OR "corticosteroide"[All Fields] OR "corticosteroides"[All Fields]) OR ("hydrocortisone"[MeSH Terms] OR "hydrocortisone"[All Fields] OR "hydrocortisones"[All Fields]) OR ("dexamethason"[All Fields] OR "dexamethasone"[MeSH Terms] OR "dexamethasone"[All Fields] OR "dexamethasone s"[All Fields] OR "dexamethasones"[All Fields]) OR ("prednison"[All Fields] OR "prednisone"[MeSH Terms] OR "prednisone"[All Fields]) OR ("prednisolon"[All Fields] OR "prednisolone"[MeSH Terms] OR "prednisolone"[All Fields]) OR ("methylprednisolone"[MeSH Terms] OR "methylprednisolone"[All Fields] OR "methylprednisolon"[All Fields]) OR ("adrenal cortex hormones"[MeSH Terms] OR ("adrenal"[All Fields] AND "cortex"[All Fields] AND "hormones"[All Fields]) OR "adrenal cortex hormones"[All Fields] OR ("adrenal"[All Fields] AND "cortex"[All Fields] AND "hormone"[All Fields]) OR "adrenal cortex hormone"[All Fields]) | 1,266,708 |
| #4 | immunosuppressive agents[Pharmacological Action] OR "immunosuppressive agents"[MeSH Terms] OR ("immunosuppressive"[All Fields] AND "agents"[All Fields]) OR "immunosuppressive agents"[All Fields] OR ("immunosuppressive"[All Fields] AND "agent"[All Fields]) OR "immunosuppressive agent"[All Fields] OR ("immunosuppressive agents"[Pharmacological Action] OR "immunosuppressive agents"[MeSH Terms] OR ("immunosuppressive"[All Fields] AND "agents"[All Fields]) OR "immunosuppressive agents"[All Fields] OR ("immunosuppressant"[All Fields] AND "agent"[All Fields]) OR "immunosuppressant agent"[All Fields]) OR "immunosuppressant*"[All Fields] | 376,816 |
| #5 | azathioprin[All Fields] OR "azathioprine"[MeSH Terms] OR "azathioprine"[All Fields] OR ("cyclophosphamide"[MeSH Terms] OR "cyclophosphamide"[All Fields] OR "cyclophosphamid"[All Fields] OR "cyclophosphamide s"[All Fields] OR "cyclophosphamides"[All Fields]) OR ("cyclosporine"[MeSH Terms] OR "cyclosporine"[All Fields] OR "ciclosporin"[All Fields] OR "ciclosporine"[All Fields] OR "cyclosporin"[All Fields] OR "cyclosporine s"[All Fields] OR "cyclosporins"[MeSH Terms] OR "cyclosporins"[All Fields] OR "cyclosporines"[All Fields]) OR ("tacrolimus"[MeSH Terms] OR "tacrolimus"[All Fields]) OR ("tacrolimus"[MeSH Terms] OR "tacrolimus"[All Fields] OR "fk506"[All Fields]) OR ("tacrolimus"[MeSH Terms] OR "tacrolimus"[All Fields] OR "fk 506"[All Fields]) OR ("mizoribin"[All Fields] OR "mizoribine"[Supplementary Concept] OR "mizoribine"[All Fields]) OR ("mizoribine"[Supplementary Concept] OR "mizoribine"[All Fields] OR "bredinin"[All Fields]) OR ("mycophenolic acid"[MeSH Terms] OR ("mycophenolic"[All Fields] AND "acid"[All Fields]) OR "mycophenolic acid"[All Fields] OR ("mycophenolate"[All Fields] AND "mofetil"[All Fields]) OR "mycophenolate mofetil"[All Fields]) OR "mycophenol*"[All Fields] OR ("mycophenolic acid"[MeSH Terms] OR ("mycophenolic"[All Fields] AND "acid"[All Fields]) OR "mycophenolic acid"[All Fields] OR "cellcept"[All Fields] OR ("mycophenolate"[All Fields] AND "mofetil"[All Fields]) OR "mycophenolate mofetil"[All Fields]) OR ("rituximab"[MeSH Terms] OR "rituximab"[All Fields] OR "rituximab s"[All Fields]) OR ("rituximab"[MeSH Terms] OR "rituximab"[All Fields] OR "rituxan"[All Fields] OR "rituximab s"[All Fields]) | 192,159 |
| #6 | nephritis[MeSH Terms] OR "nephritis"[All Fields] OR "nephritides"[All Fields] OR ("glomerulonephritis"[MeSH Terms] OR "glomerulonephritis"[All Fields] OR "glomerulonephritides"[All Fields]) OR ("kidney diseases"[MeSH Terms] OR ("kidney"[All Fields] AND "diseases"[All Fields]) OR "kidney diseases"[All Fields] OR "nephropathies"[All Fields] OR "nephropathy"[All Fields]) OR ("kidney diseases"[MeSH Terms] OR ("kidney"[All Fields] AND "diseases"[All Fields]) OR "kidney diseases"[All Fields] OR ("kidney"[All Fields] AND "disease"[All Fields]) OR "kidney disease"[All Fields]) OR ("kidney diseases"[MeSH Terms] OR ("kidney"[All Fields] AND "diseases"[All Fields]) OR "kidney diseases"[All Fields] OR ("renal"[All Fields] AND "disease"[All Fields]) OR "renal disease"[All Fields]) OR ("renal"[All Fields] OR "renals"[All Fields]) OR ("kidney"[MeSH Terms] OR "kidney"[All Fields] OR "kidneys"[All Fields] OR "kidney s"[All Fields]) | 1,273,967 |
| #7 | (#1 OR #2) AND (#3 OR #4 OR #5) AND #6 | 1,053 |

**10-2. Is cyclosporine recommended for severe cases of pediatric IgA vasculitis nephritis?**

Search date：9-Nov-2021

| No. | Search expression | Number of searches |
| --- | --- | --- |
| #1 | "purpura, schoenlein henoch"[MeSH Terms] OR ("purpura"[All Fields] AND "schoenlein henoch"[All Fields]) OR "schoenlein-henoch purpura"[All Fields] OR ("purpura"[All Fields] AND "schoenlein"[All Fields] AND "henoch"[All Fields]) OR "purpura schoenlein henoch"[All Fields] OR ("purpura, schoenlein henoch"[MeSH Terms] OR ("purpura"[All Fields] AND "schoenlein henoch"[All Fields]) OR "schoenlein-henoch purpura"[All Fields] OR ("purpura"[All Fields] AND "schonlein"[All Fields] AND "henoch"[All Fields]) OR "purpura schonlein henoch"[All Fields]) OR ("purpura, schoenlein henoch"[MeSH Terms] OR ("purpura"[All Fields] AND "schoenlein henoch"[All Fields]) OR "schoenlein-henoch purpura"[All Fields] OR "immunoglobulin a vasculitis"[All Fields]) OR ("IgA"[All Fields] AND ("vasculitide"[All Fields] OR "vasculities"[All Fields] OR "vasculitis"[MeSH Terms] OR "vasculitis"[All Fields] OR "vasculitides"[All Fields])) OR ("purpura, schoenlein henoch"[MeSH Terms] OR ("purpura"[All Fields] AND "schoenlein henoch"[All Fields]) OR "schoenlein-henoch purpura"[All Fields] OR ("allergic"[All Fields] AND "purpura"[All Fields]) OR "allergic purpura"[All Fields]) OR ("purpura, schoenlein henoch"[MeSH Terms] OR ("purpura"[All Fields] AND "schoenlein henoch"[All Fields]) OR "schoenlein-henoch purpura"[All Fields] OR ("anaphylactoid"[All Fields] AND "purpura"[All Fields]) OR "anaphylactoid purpura"[All Fields]) OR (("anaphylactic"[All Fields] OR "anaphylactically"[All Fields]) AND ("purpura"[MeSH Terms] OR "purpura"[All Fields] OR "purpuras"[All Fields])) | 7127 |
| #2 | ("purpura"[MeSH Terms] OR "purpura"[All Fields] OR "purpuras"[All Fields]) AND ("nephritis"[MeSH Terms] OR "nephritis"[All Fields] OR "nephritides"[All Fields]) | 2178 |
| #3 | cyclosporine[MeSH Terms] OR "cyclosporine"[All Fields] OR "ciclosporin"[All Fields] OR "ciclosporine"[All Fields] OR "cyclosporin"[All Fields] OR "cyclosporine s"[All Fields] OR "cyclosporins"[MeSH Terms] OR "cyclosporins"[All Fields] OR "cyclosporines"[All Fields] OR ("cyclosporine"[MeSH Terms] OR "cyclosporine"[All Fields] OR "ciclosporin"[All Fields] OR "ciclosporine"[All Fields] OR "cyclosporin"[All Fields] OR "sandimmun"[All Fields] OR "sandimmune"[All Fields] OR "cyclosporine s"[All Fields] OR "cyclosporins"[MeSH Terms] OR "cyclosporins"[All Fields] OR "cyclosporines"[All Fields]) OR ("cyclosporine"[MeSH Terms] OR "cyclosporine"[All Fields] OR "ciclosporin"[All Fields] OR "ciclosporine"[All Fields] OR "cyclosporin"[All Fields] OR "neoral"[All Fields] OR "cyclosporine s"[All Fields] OR "cyclosporins"[MeSH Terms] OR "cyclosporins"[All Fields] OR "cyclosporines"[All Fields]) OR ("calcineurin inhibitors"[Pharmacological Action] OR "calcineurin inhibitors"[MeSH Terms] OR ("calcineurin"[All Fields] AND "inhibitors"[All Fields]) OR "calcineurin inhibitors"[All Fields] OR ("calcineurin"[All Fields] AND "inhibitor"[All Fields]) OR "calcineurin inhibitor"[All Fields]) | 78231 |
| #4 | (#1 OR #2) AND #3 | 79 |
| #5 | #4 AND ("1966/01/01"[EDAT]:"2021/07/31"[EDAT]) | 77 |

**CQ11: Is methylprednisolone pulse therapy recommended for severe cases of pediatric IgA vasculitis nephritis?**

Search date：23-Nov-2021

| No. | Search expression | Number of searches |
| --- | --- | --- |
| #1 | "purpura, schoenlein henoch"[MeSH Terms] OR ("purpura"[All Fields] AND "schoenlein henoch"[All Fields]) OR "schoenlein-henoch purpura"[All Fields] OR ("purpura"[All Fields] AND "schoenlein"[All Fields] AND "henoch"[All Fields]) OR "purpura schoenlein henoch"[All Fields] OR ("purpura, schoenlein henoch"[MeSH Terms] OR ("purpura"[All Fields] AND "schoenlein henoch"[All Fields]) OR "schoenlein-henoch purpura"[All Fields] OR ("purpura"[All Fields] AND "schonlein"[All Fields] AND "henoch"[All Fields]) OR "purpura schonlein henoch"[All Fields]) OR ("purpura, schoenlein henoch"[MeSH Terms] OR ("purpura"[All Fields] AND "schoenlein henoch"[All Fields]) OR "schoenlein-henoch purpura"[All Fields] OR "immunoglobulin a vasculitis"[All Fields]) OR ("IgA"[All Fields] AND ("vasculitide"[All Fields] OR "vasculities"[All Fields] OR "vasculitis"[MeSH Terms] OR "vasculitis"[All Fields] OR "vasculitides"[All Fields])) OR ("purpura, schoenlein henoch"[MeSH Terms] OR ("purpura"[All Fields] AND "schoenlein henoch"[All Fields]) OR "schoenlein-henoch purpura"[All Fields] OR ("allergic"[All Fields] AND "purpura"[All Fields]) OR "allergic purpura"[All Fields]) OR ("purpura, schoenlein henoch"[MeSH Terms] OR ("purpura"[All Fields] AND "schoenlein henoch"[All Fields]) OR "schoenlein-henoch purpura"[All Fields] OR ("anaphylactoid"[All Fields] AND "purpura"[All Fields]) OR "anaphylactoid purpura"[All Fields]) OR (("anaphylactic"[All Fields] OR "anaphylactically"[All Fields]) AND ("purpura"[MeSH Terms] OR "purpura"[All Fields] OR "purpuras"[All Fields])) | 7145 |
| #2 | ("purpura"[MeSH Terms] OR "purpura"[All Fields] OR "purpuras"[All Fields]) AND ("nephritis"[MeSH Terms] OR "nephritis"[All Fields] OR "nephritides"[All Fields]) | 2179 |
| #3 | "steroidal"[All Fields] OR "steroidals"[All Fields] OR "steroidic"[All Fields] OR "steroids"[MeSH Terms] OR "steroids"[All Fields] OR "steroid"[All Fields] OR ("glucocorticoids"[Pharmacological Action] OR "glucocorticoids"[MeSH Terms] OR "glucocorticoids"[All Fields] OR "glucocorticoid"[All Fields]) OR ("adrenal cortex hormones"[MeSH Terms] OR ("adrenal"[All Fields] AND "cortex"[All Fields] AND "hormones"[All Fields]) OR "adrenal cortex hormones"[All Fields] OR "corticosteroid"[All Fields] OR "corticosteroids"[All Fields] OR "corticosteroidal"[All Fields] OR "corticosteroide"[All Fields] OR "corticosteroides"[All Fields]) OR ("hydrocortisone"[MeSH Terms] OR "hydrocortisone"[All Fields] OR "hydrocortisones"[All Fields]) OR ("dexamethason"[All Fields] OR "dexamethasone"[MeSH Terms] OR "dexamethasone"[All Fields] OR "dexamethasone s"[All Fields] OR "dexamethasones"[All Fields]) OR ("prednison"[All Fields] OR "prednisone"[MeSH Terms] OR "prednisone"[All Fields]) OR ("prednisolon"[All Fields] OR "prednisolone"[MeSH Terms] OR "prednisolone"[All Fields]) OR ("methylprednisolone"[MeSH Terms] OR "methylprednisolone"[All Fields] OR "methylprednisolon"[All Fields]) OR ("adrenal cortex hormones"[MeSH Terms] OR ("adrenal"[All Fields] AND "cortex"[All Fields] AND "hormones"[All Fields]) OR "adrenal cortex hormones"[All Fields] OR ("adrenal"[All Fields] AND "cortex"[All Fields] AND "hormone"[All Fields]) OR "adrenal cortex hormone"[All Fields]) | 1272224 |
| #4 | ("steroidal"[All Fields] OR "steroidals"[All Fields] OR "steroidic"[All Fields] OR "steroids"[MeSH Terms] OR "steroids"[All Fields] OR "steroid"[All Fields] OR ("glucocorticoids"[Pharmacological Action] OR "glucocorticoids"[MeSH Terms] OR "glucocorticoids"[All Fields] OR "glucocorticoid"[All Fields]) OR ("adrenal cortex hormones"[MeSH Terms] OR ("adrenal"[All Fields] AND "cortex"[All Fields] AND "hormones"[All Fields]) OR "adrenal cortex hormones"[All Fields] OR "corticosteroid"[All Fields] OR "corticosteroids"[All Fields] OR "corticosteroidal"[All Fields] OR "corticosteroide"[All Fields] OR "corticosteroides"[All Fields]) OR ("hydrocortisone"[MeSH Terms] OR "hydrocortisone"[All Fields] OR "hydrocortisones"[All Fields]) OR ("dexamethason"[All Fields] OR "dexamethasone"[MeSH Terms] OR "dexamethasone"[All Fields] OR "dexamethasone s"[All Fields] OR "dexamethasones"[All Fields]) OR ("prednison"[All Fields] OR "prednisone"[MeSH Terms] OR "prednisone"[All Fields]) OR ("prednisolon"[All Fields] OR "prednisolone"[MeSH Terms] OR "prednisolone"[All Fields]) OR ("methylprednisolone"[MeSH Terms] OR "methylprednisolone"[All Fields] OR "methylprednisolon"[All Fields]) OR ("adrenal cortex hormones"[MeSH Terms] OR ("adrenal"[All Fields] AND "cortex"[All Fields] AND "hormones"[All Fields]) OR "adrenal cortex hormones"[All Fields] OR ("adrenal"[All Fields] AND "cortex"[All Fields] AND "hormone"[All Fields]) OR "adrenal cortex hormone"[All Fields])) AND "pulse*"[All Fields] | 17040 |
| #5 | "methylprednisolone"[MeSH Terms] OR "methylprednisolone"[All Fields] OR "methylprednisolon"[All Fields] | 28590 |
| #6 | ("steroidal"[All Fields] OR "steroidals"[All Fields] OR "steroidic"[All Fields] OR "steroids"[MeSH Terms] OR "steroids"[All Fields] OR "steroid"[All Fields]) AND "pulse*"[All Fields] | 14057 |
| #7 | pulse therapy*[All Fields] OR "pulse therapy, drug"[MeSH Terms] | 143274 |
| #8 | "high"[All Fields] AND "dose"[All Fields] AND ("steroidal"[All Fields] OR "steroidals"[All Fields] OR "steroidic"[All Fields] OR "steroids"[MeSH Terms] OR "steroids"[All Fields] OR "steroid"[All Fields]) | 34834 |
| #9 | (#1 OR #2) AND (#4 OR #5 OR #6 OR #7 OR #8) | 321 |
| #10 | "nephritis"[MeSH Terms] OR "nephritis"[All Fields] OR "nephritides"[All Fields] OR ("glomerulonephritis"[MeSH Terms] OR "glomerulonephritis"[All Fields] OR "glomerulonephritides"[All Fields]) OR ("kidney diseases"[MeSH Terms] OR ("kidney"[All Fields] AND "diseases"[All Fields]) OR "kidney diseases"[All Fields] OR "nephropathies"[All Fields] OR "nephropathy"[All Fields]) OR ("kidney diseases"[MeSH Terms] OR ("kidney"[All Fields] AND "diseases"[All Fields]) OR "kidney diseases"[All Fields] OR ("kidney"[All Fields] AND "disease"[All Fields]) OR "kidney disease"[All Fields]) OR ("kidney diseases"[MeSH Terms] OR ("kidney"[All Fields] AND "diseases"[All Fields]) OR "kidney diseases"[All Fields] OR ("renal"[All Fields] AND "disease"[All Fields]) OR "renal disease"[All Fields]) OR ("renal"[All Fields] OR "renals"[All Fields]) OR ("kidney"[MeSH Terms] OR "kidney"[All Fields] OR "kidneys"[All Fields] OR "kidney s"[All Fields]) | 1280289 |
| #11 | #9 AND #10 | 237 |

**CQ12: Is pulse urokinase therapy recommended for severe cases of pediatric IgAVN?**

Search date：25-Jan-2022

| No. | Search expression | Number of searches |
| --- | --- | --- |
| #1 | "purpura, schoenlein henoch"[MeSH Terms] OR ("purpura"[All Fields] AND "schoenlein henoch"[All Fields]) OR "schoenlein-henoch purpura"[All Fields] OR ("purpura"[All Fields] AND "schoenlein"[All Fields] AND "henoch"[All Fields]) OR "purpura schoenlein henoch"[All Fields] OR ("purpura, schoenlein henoch"[MeSH Terms] OR ("purpura"[All Fields] AND "schoenlein henoch"[All Fields]) OR "schoenlein-henoch purpura"[All Fields] OR ("purpura"[All Fields] AND "schonlein"[All Fields] AND "henoch"[All Fields]) OR "purpura schonlein henoch"[All Fields]) OR ("purpura, schoenlein henoch"[MeSH Terms] OR ("purpura"[All Fields] AND "schoenlein henoch"[All Fields]) OR "schoenlein-henoch purpura"[All Fields] OR "immunoglobulin a vasculitis"[All Fields]) OR ("IgA"[All Fields] AND ("vasculitide"[All Fields] OR "vasculities"[All Fields] OR "vasculitis"[MeSH Terms] OR "vasculitis"[All Fields] OR "vasculitides"[All Fields])) OR ("purpura, schoenlein henoch"[MeSH Terms] OR ("purpura"[All Fields] AND "schoenlein henoch"[All Fields]) OR "schoenlein-henoch purpura"[All Fields] OR ("allergic"[All Fields] AND "purpura"[All Fields]) OR "allergic purpura"[All Fields]) OR ("purpura, schoenlein henoch"[MeSH Terms] OR ("purpura"[All Fields] AND "schoenlein henoch"[All Fields]) OR "schoenlein-henoch purpura"[All Fields] OR ("anaphylactoid"[All Fields] AND "purpura"[All Fields]) OR "anaphylactoid purpura"[All Fields]) OR (("anaphylactic"[All Fields] OR "anaphylactically"[All Fields]) AND ("purpura"[MeSH Terms] OR "purpura"[All Fields] OR "purpuras"[All Fields])) | 6,989 |
| #2 | ("purpura"[MeSH Terms] OR "purpura"[All Fields] OR "purpuras"[All Fields]) AND ("nephritis"[MeSH Terms] OR "nephritis"[All Fields] OR "nephritides"[All Fields]) | 2,187 |
| #3 | urokinase type plasminogen activator[MeSH Terms] OR ("urokinase type"[All Fields] AND "plasminogen"[All Fields] AND "activator"[All Fields]) OR "urokinase type plasminogen activator"[All Fields] OR "urokinase"[All Fields] OR "urokinases"[All Fields] | 18,848 |
| #4 | (#1 OR #2) AND #3 | 17 |

**CQ13: Is plasma exchange recommended for severe cases of pediatric IgA vasculitis nephritis?**

Search date：30-Oct-2021

| No. | Search expression | Number of searches |
| --- | --- | --- |
| #1 | "purpura, schoenlein henoch"[MeSH Terms] OR ("purpura"[All Fields] AND "schoenlein henoch"[All Fields]) OR "schoenlein-henoch purpura"[All Fields] OR ("purpura"[All Fields] AND "schoenlein"[All Fields] AND "henoch"[All Fields]) OR "purpura schoenlein henoch"[All Fields] OR ("purpura, schoenlein henoch"[MeSH Terms] OR ("purpura"[All Fields] AND "schoenlein henoch"[All Fields]) OR "schoenlein-henoch purpura"[All Fields] OR ("purpura"[All Fields] AND "schonlein"[All Fields] AND "henoch"[All Fields]) OR "purpura schonlein henoch"[All Fields]) OR ("purpura, schoenlein henoch"[MeSH Terms] OR ("purpura"[All Fields] AND "schoenlein henoch"[All Fields]) OR "schoenlein-henoch purpura"[All Fields] OR "immunoglobulin a vasculitis"[All Fields]) OR ("IgA"[All Fields] AND ("vasculitide"[All Fields] OR "vasculities"[All Fields] OR "vasculitis"[MeSH Terms] OR "vasculitis"[All Fields] OR "vasculitides"[All Fields])) OR ("purpura, schoenlein henoch"[MeSH Terms] OR ("purpura"[All Fields] AND "schoenlein henoch"[All Fields]) OR "schoenlein-henoch purpura"[All Fields] OR ("allergic"[All Fields] AND "purpura"[All Fields]) OR "allergic purpura"[All Fields]) OR ("purpura, schoenlein henoch"[MeSH Terms] OR ("purpura"[All Fields] AND "schoenlein henoch"[All Fields]) OR "schoenlein-henoch purpura"[All Fields] OR ("anaphylactoid"[All Fields] AND "purpura"[All Fields]) OR "anaphylactoid purpura"[All Fields]) OR (("anaphylactic"[All Fields] OR "anaphylactically"[All Fields]) AND ("purpura"[MeSH Terms] OR "purpura"[All Fields] OR "purpuras"[All Fields])) | 7,100 |
| #2 | ("purpura"[MeSH Terms] OR "purpura"[All Fields] OR "purpuras"[All Fields]) AND ("nephritis"[MeSH Terms] OR "nephritis"[All Fields] OR "nephritides"[All Fields]) | 2,165 |
| #3 | "plasmapheresis"[MeSH Terms] OR "plasmapheresis"[All Fields] OR ("plasma"[All Fields] AND "exchange"[All Fields]) OR "plasma exchange"[All Fields] OR "plasma exchange"[MeSH Terms] OR ("plasma"[All Fields] AND "exchange"[All Fields]) OR ("blood component removal"[MeSH Terms] OR ("blood"[All Fields] AND "component"[All Fields] AND "removal"[All Fields]) OR "blood component removal"[All Fields] OR "apheresis"[All Fields]) OR (("plasma"[MeSH Terms] OR "plasma"[All Fields] OR "plasmas"[All Fields] OR "plasma s"[All Fields]) AND ("infusate"[All Fields] OR "infusates"[All Fields] OR "infuse"[All Fields] OR "infused"[All Fields] OR "infuser"[All Fields] OR "infusers"[All Fields] OR "infuses"[All Fields] OR "infusing"[All Fields] OR "infusion"[All Fields] OR "infusions"[All Fields])) OR "plasmapharesis"[All Fields] | 108,172 |
| #4 | (#1 OR #2) AND #3 | 299 |
| #5 | nephritis[MeSH Terms] OR "nephritis"[All Fields] OR "nephritides"[All Fields] OR ("glomerulonephritis"[MeSH Terms] OR "glomerulonephritis"[All Fields] OR "glomerulonephritides"[All Fields]) OR ("kidney diseases"[MeSH Terms] OR ("kidney"[All Fields] AND "diseases"[All Fields]) OR "kidney diseases"[All Fields] OR "nephropathies"[All Fields] OR "nephropathy"[All Fields]) OR ("kidney diseases"[MeSH Terms] OR ("kidney"[All Fields] AND "diseases"[All Fields]) OR "kidney diseases"[All Fields] OR ("kidney"[All Fields] AND "disease"[All Fields]) OR "kidney disease"[All Fields]) OR ("kidney diseases"[MeSH Terms] OR ("kidney"[All Fields] AND "diseases"[All Fields]) OR "kidney diseases"[All Fields] OR ("renal"[All Fields] AND "disease"[All Fields]) OR "renal disease"[All Fields]) OR ("renal"[All Fields] OR "renals"[All Fields]) OR ("kidney"[MeSH Terms] OR "kidney"[All Fields] OR "kidneys"[All Fields] OR "kidney s"[All Fields]) | 1,273,967 |
| #6 | #4 AND #5 | 220 |

**CQ14: Is the combination of tonsillectomy and steroid pulse therapy recommended for severe cases of pediatric IgA vasculitis nephritis?**

Search date：14-Nov-2021

| No. | Search expression | Number of searches |
| --- | --- | --- |
| #1 | "purpura, schoenlein henoch"[MeSH Terms] OR ("purpura"[All Fields] AND "schoenlein henoch"[All Fields]) OR "schoenlein-henoch purpura"[All Fields] OR ("purpura"[All Fields] AND "schoenlein"[All Fields] AND "henoch"[All Fields]) OR "purpura schoenlein henoch"[All Fields] OR ("purpura, schoenlein henoch"[MeSH Terms] OR ("purpura"[All Fields] AND "schoenlein henoch"[All Fields]) OR "schoenlein-henoch purpura"[All Fields] OR ("purpura"[All Fields] AND "schonlein"[All Fields] AND "henoch"[All Fields]) OR "purpura schonlein henoch"[All Fields]) OR ("purpura, schoenlein henoch"[MeSH Terms] OR ("purpura"[All Fields] AND "schoenlein henoch"[All Fields]) OR "schoenlein-henoch purpura"[All Fields] OR "immunoglobulin a vasculitis"[All Fields]) OR ("IgA"[All Fields] AND ("vasculitide"[All Fields] OR "vasculities"[All Fields] OR "vasculitis"[MeSH Terms] OR "vasculitis"[All Fields] OR "vasculitides"[All Fields])) OR ("purpura, schoenlein henoch"[MeSH Terms] OR ("purpura"[All Fields] AND "schoenlein henoch"[All Fields]) OR "schoenlein-henoch purpura"[All Fields] OR ("allergic"[All Fields] AND "purpura"[All Fields]) OR "allergic purpura"[All Fields]) OR ("purpura, schoenlein henoch"[MeSH Terms] OR ("purpura"[All Fields] AND "schoenlein henoch"[All Fields]) OR "schoenlein-henoch purpura"[All Fields] OR ("anaphylactoid"[All Fields] AND "purpura"[All Fields]) OR "anaphylactoid purpura"[All Fields]) OR (("anaphylactic"[All Fields] OR "anaphylactically"[All Fields]) AND ("purpura"[MeSH Terms] OR "purpura"[All Fields] OR "purpuras"[All Fields])) | 7147 |
| #2 | ("purpura"[MeSH Terms] OR "purpura"[All Fields] OR "purpuras"[All Fields]) AND ("nephritis"[MeSH Terms] OR "nephritis"[All Fields] OR "nephritides"[All Fields]) | 2179 |
| #3 | "tonsillectomy"[MeSH Terms] OR "tonsillectomy"[All Fields] OR "tonsillectomies"[All Fields] | 12775 |
| #4 | "steroidal"[All Fields] OR "steroidals"[All Fields] OR "steroidic"[All Fields] OR "steroids"[MeSH Terms] OR "steroids"[All Fields] OR "steroid"[All Fields] OR ("glucocorticoids"[Pharmacological Action] OR "glucocorticoids"[MeSH Terms] OR "glucocorticoids"[All Fields] OR "glucocorticoid"[All Fields]) OR ("adrenal cortex hormones"[MeSH Terms] OR ("adrenal"[All Fields] AND "cortex"[All Fields] AND "hormones"[All Fields]) OR "adrenal cortex hormones"[All Fields] OR "corticosteroid"[All Fields] OR "corticosteroids"[All Fields] OR "corticosteroidal"[All Fields] OR "corticosteroide"[All Fields] OR "corticosteroides"[All Fields]) OR ("hydrocortisone"[MeSH Terms] OR "hydrocortisone"[All Fields] OR "hydrocortisones"[All Fields]) OR ("dexamethason"[All Fields] OR "dexamethasone"[MeSH Terms] OR "dexamethasone"[All Fields] OR "dexamethasone s"[All Fields] OR "dexamethasones"[All Fields]) OR ("prednison"[All Fields] OR "prednisone"[MeSH Terms] OR "prednisone"[All Fields]) OR ("prednisolon"[All Fields] OR "prednisolone"[MeSH Terms] OR "prednisolone"[All Fields]) OR ("methylprednisolone"[MeSH Terms] OR "methylprednisolone"[All Fields] OR "methylprednisolon"[All Fields]) OR ("adrenal cortex hormones"[MeSH Terms] OR ("adrenal"[All Fields] AND "cortex"[All Fields] AND "hormones"[All Fields]) OR "adrenal cortex hormones"[All Fields] OR ("adrenal"[All Fields] AND "cortex"[All Fields] AND "hormone"[All Fields]) OR "adrenal cortex hormone"[All Fields]) | 1272524 |
| #5 | (#1 OR #2) AND #3 AND #4 | 17 |
